# Molecular and morphological data of the freshwater fish *Glandulocauda melanopleura *(Characiformes: Characidae) provide evidences of river captures and local differentiation in the Brazilian Atlantic Forest

**DOI:** 10.1371/journal.pone.0194247

**Published:** 2018-03-26

**Authors:** Priscila Camelier, Naércio Aquino Menezes, Guilherme José Costa-Silva, Claudio Oliveira

**Affiliations:** 1 Museu de Zoologia da Universidade de São Paulo, CEP, São Paulo, SP, Brazil; 2 Universidade Santo Amaro, Rua Prof. Enéas de Siqueira Neto, Jardim das Imbuias, CEP, São Paulo, SP, Brazil; 3 Laboratório de Biologia e Genética de Peixes, Departamento de Morfologia, Instituto de Biociências, Universidade Estadual Paulista, Distrito Rubião Jr., CEP, Botucatu, SP, Brazil; University of Innsbruck, AUSTRIA

## Abstract

The current distribution of freshwater fishes across multiple basins along Eastern Brazil can be associated to two main events: river captures or temporary paleoconnections. Apparently, river captures had a more significant role on distribution and structuring of species from upland areas, such as *Glandulocauda melanopleura*. Populations of this species are found in contiguous drainages in presently isolated upper parts of Rio Tietê and the coastal basins of Guaratuba, Itatinga, Itanháem, and Ribeira de Iguape, in the Atlantic Forest domain. The allopatric and disjoint distribution of *G*. *melanopleura* associated with variation of morphological characters detected among geographically isolated populations stimulated this study. Thus, an integrative approach was undertaken, including morphological and molecular data, to better understand the evolutionary history of the species and the area where it occurs. Molecular analyses based on two mitochondrial markers revealed a strong genetic structure within *G*. *melanopleura*, that allowed recognition of two lineages, one distributed in both the upper Tietê and Itanhaém and the other in the Guaratuba. Overall, morphological data revealed some intraspecific overlapping variation, indicating that all samples are conspecific. Phylogenetic and phylogeographic analyses allied to divergence times and geomorphological information indicate that the current distribution of *G*. *melanopleura* is a result of relatively recent river captures involving the Tietê and some other coastal drainages. Although of recent origin, they occurred long enough to completely isolate these populations, since there are no haplotypes sharing between them. The conservation status of this species is also discussed, and our results corroborate the need to understand population structure for conservation planning.

## Introduction

The tribe Glandulocaudini (former Glandulocaudinae of Menezes & Weitzman [1]) includes the genera *Lophiobrycon*, *Glandulocauda*, and *Mimagoniates* [1,2], represented by 10 small species, distributed in freshwater habitats of eastern and southern Brazil, Paraguay, and northeastern Uruguay [1,3]. Their degree of morphological and behavioral specialization, as well as distributional patterns are of great importance for evolutionary studies and to understand biogeographical patterns of South American freshwater fishes (e.g., [3–8]).

The genus *Glandulocauda* was created by Eigenmann [9] to include *G*. *inequalis*, *G*. *melanogenys* (type species), and *G*. *melanopleura*, defined by a combination of morphological characters including premaxillary teeth in two distinct series, with four, rarely five, teeth in the inner series; third infraorbital covering the entire cheek; caudal fin naked, with a few scales forming a flap on the base of the rays just above the middle of the fin; and dorsal-fin origin nearer middle caudal fin than to snout ([9]: 168–170). However, its present composition is quite different from the original one. *Glandulocauda inequalis* was placed in *Mimagoniates* [10,11] and, more recently, Menezes and Weitzman [1], in reviewing the systematics of the Glandulocaudini (at the time Glandulocaudinae), considered *G*. *melanogenys* a junior synonym of *Hyphessobrycon melanopleurus* Ellis ([12]: 157–158), which led these authors to propose the replacement of the species name *melanogenys* Eigenmann by *melanopleura* Ellis. However, this taxonomic change caused *G*. *melanopleura* as proposed by Eigenmann [9] to become a junior secondary homonym of *G*. *melanopleura* of Ellis (1911) because both species were kept in the same genus, *Glandulocauda* [1]. To resolve this issue, these authors proposed a new replacement name, *G*. *caerulea* Menezes & Weitzman, for *G*. *melanopleura* Eigenmann. Thus, currently, there are two valid species of *Glandulocauda*: *G*. *caerulea* Menezes & Weitzman and *G*. *melanopleura* (Ellis), and our study focus on this latter species, which is the type-species of the genus.

*Glandulocauda melanopleura* was described based on specimens from a headwater stream of the Rio Tietê drainage, the main tributary of the left bank of the Paraná river basin, state of São Paulo, Southeastern Brazil, and for a long time it was considered endemic to this basin (e.g., [13]). However, populations of *G*. *melanopleura* were latter also reported from some other streams, tributaries of coastal rivers adjoining the upper Paraná basin, which drain directly to the Atlantic Ocean along the Serra do Mar coastal range of SE Brazil: upper portions of the Guaratuba [5], Itatinga [14], and Juquiá (a tributary of the upper Rio Ribeira de Iguape) river basins [1]. Finally, during recent expeditions to the upper portion of the headwaters of Rio Itanhaém drainage, a relatively large coastal river in SE Brazil, at São Paulo, one more isolated population of *G*. *melanopleura* was found. This represents the fourth record of the species outside the upper Tietê drainage. The streams where *G*. *melanopleura* occurs are currently isolated from each other and are characterized mainly by draining areas of high altitudes (about 800 m above sea level) of the Brazilian crystalline shield in the Atlantic Forest domain [1,3,5], one of the most important and threatened biodiversity hotspots of the world [15,16]. The stretches of the Serra do Mar escarpment in which *G*. *melanopleura* occurs are areas remarkably unstable, subject to heavy rains and large landslides [5], and the recognized intense tectonic activity in this region has been acting on the evolutionary history of both the river basins and their ichthyofauna [3–6,8].

In their above-mentioned morphological review of glandulocaudines, Menezes & Weitzman [1] also examined specimens that represent new records of *G*. *melanopleura* outside the upper Tietê basin and reported variation of some morphological characters among these populations (e.g., number of anal-fin rays, scales around caudal peduncle, and a few other meristic counts), some of which even overlap with those of *G*. *caerulea*. Since the specimens they examined were very similar to *G*. *melanopleura* with respect to most other meristic and morphometric characters, they considered all samples conspecific and suggested that a more detailed analysis of character variation within the range of this species should be performed. The variation of morphological characters detected among geographically isolated populations of *G*. *melanopleura* combined with its allopatric distribution in basins currently isolated motivated the present study. Although studies on systematics and biogeography based on morphological grounds improved the knowledge of the Glandulocaudini, especially regarding *G*. *melanopleura*, molecular evidence was never contemplated. In this paper, an extensive population analysis of *G*. *melanopleura* was undertaken, based on an integrative approach and including both morphological and molecular data, to better understand the evolutionary history of the species. *Glandulocauda melanopleura* distributional pattern and the geomorphological processes and events that are involved with its generation are discussed. We think that the results obtained will be useful for conservation planning not only of *G*. *melanopleura* but also of other freshwater fish species from streams draining the Atlantic Forest domain.

## Material and methods

### Ethical statement

We declare that the fish under study are not protected under wildlife conservation, and no experimentation was conducted on live specimens. All specimens used were collected in accordance with Brazilian laws, and the sampling was approved by the Instituto Chico Mendes de Conservação da Biodiversidade (ICMBio) and Sistema de Autorização e Informação em Biodiversidade (SISBIO) under a license issued in the name of Dr. Osvaldo Oyakawa, research specialist of the fish section of the Museu de Zoologia da Universidade de São Paulo, where this study was carried out (SISBIO number 21924–1). After collection, the animals were anesthetized and sacrificed using 1% benzocaine in water as approved by the Bioscience Institute/UNESP Ethics Committee on the Use of Animals (CEUA; protocol 405) and recommended by the National Council for the Control of Animal Experimentation and the Federal Board of Veterinary Medicine.

### Molecular analyses

#### Taxon sampling, DNA extraction, and sequencing

Tissue samples from 74 specimens, representing 20 individuals of *G*. *melanopleura* (Table 1) and 54 of other six species of Glandulocaudini (*G*. *caerulea*, *M*. *inequalis*, *M*. *lateralis*, *M*. *microlepis*, *M*. *rheocharis*, and *M*. *sylvicola*, S1 Table) were obtained from fish collections or field expeditions carried out between 2012 and 2015. All the species collected are deposited in the Laboratório de Biologia e Genética de Peixes (LBP), Departamento de Morfologia, Instituto de Biociências, Universidade Estadual Paulista, Botucatu, São Paulo, and the Museu de Zoologia da Universidade de São Paulo (MZUSP), São Paulo, Brazil. Samples of *G*. *melanopleura* include individuals from most river systems throughout the range of this species: upper Rio Tietê, Rio Guaratuba, and Rio Itanhaém. Besides these, taxon sampling also included *Lophiobrycon weitzmani*, representatives of all the other stevardiin tribes (i.e., Creagrutini, Diapomini, Eretmobryconini, Hemibryconini, Stevardiini, and Xenurobryconini *sensu* Thomaz *et al*. [2]) plus three non-stevardiin species: *Bryconops caudomaculatus*, *Cheirodon ibicuhiensis*, and *Spintherobolus leptoura*. Sequences of all non-glandulocaudin species plus *L*. *weitzmani* were obtained from the GenBank database deposited by Oliveira *et al*. [17], Pereira *et al*. [18], or Thomaz *et al*. [2]. All glandulocaudin vouchers, including *L*. *weitzmani*, were identified to species level based on diagnostic morphological traits. Species used in the phylogenetic analyses, identification codes of samples, vouchers, and GenBank accession numbers are given in S1 Table. Institutional abbreviations follow Fricke & Eschmeyer [19], with inclusion of the tissue collection of the Museu de Zoologia da Universidade de São Paulo (MZict).

**Table 1:** Tissues samples of *Glandulocauda melanopleura* used in this study.

Population/Drainage	Lot number	Vouchers	Locality
Upper Tietê/Paraná	LBP 4507	LBP 24537, 24538, 24539, 24540, 24541, 24542, 24553	Rio Paranapiacaba, Santo André, 23°40’13.2”S, 46°18’39.6”W, 787 m
Guaratuba/Coastal	MZUSP 115244	MZict 2067, 2069, 2070	Rio Guaratuba, Bertioga, 23°40’05.0”S, 45°53’57.1”W, 812 m
Itanhaém/Coastal	MZUSP 111017	LBP 70058, 70059, 70060, 70061, 70062, 70063, 70064, 70065 70066, 70067	Rio Capivari, tributary of Rio Branco, Itanhaém, 23°59’19.4”S, 46°40’50.4”W, 734 m

Populations, lot number, vouchers, and locality information including geographical coordinates and altitudes. All samples from São Paulo state, Brazil. [GenBank accession number ranges: 16S = MG958088-MG958089/MH036146-MH036163; COI = MG967568-MG967569/MH036036-MH036053].

Total genomic DNA was extracted from muscle and fin tissues preserved in 96% ethanol with a DNeasy Tissue Kit (Qiagen), according to instructions of the manufacturer. Partial sequences of the mitochondrial genes *16S rRNA* and cytochrome c oxidase subunit I (*COI*) were amplified by polymerase chain reaction (PCR) with the primers described by Palumbi [20] and Ward *et al*. [21], respectively. Amplifications were performed in a total volume of 12.5 μl, with 1.25 μl of 10X buffer (10 mM Tris-HCl+15 mM MgCl_2_), 0.375 μl MgCl_2_ (50 nM), 0.5 μl dNTPs (200 nM of each), 0.25 μl each 5 mM primer, 0.05 μl Platinum *Taq* Polymerase (Invitrogen), 9.075 μl of double-distilled water, and 1 μl template DNA (12 ng). The thermo-cycler profile consisted of an initial denaturation step at 95°C for 5 min; followed by 35 cycles of chain denaturation (45 s at 95°C), annealing (30 s at 52°C for *16S* and 54°C for *COI*), and nucleotide extension (1 min at 72°C); plus a final extension step at 72°C for 7 min. The PCR products were first visually identified on a 1% agarose gel and then purified using ExoSap-IT® (USB Corporation) following the instructions of the manufacturer. The purified PCR products were sequenced using the Big DyeTM Terminator v 3.1 Cycle Sequencing Ready Reaction Kit (Applied Biosystems), purified again by ethanol precipitation and loaded on an automatic sequencer 3130-Genetic Analyzer (Applied Biosystems) in the Instituto de Biociências, Universidade Estadual Paulista, Botucatu, São Paulo, Brazil. All sequences were read twice (forward and reverse). All sequences produced in this study were deposited in the GenBank.

#### Alignment, phylogenetic and Generalized Mixed Yule-Coalescent analyses, and estimation of divergence times

Electropherograms were inspected and assembled in contigs from forward and reverse strands using Geneious v. 4.8.5 [22]. Sequences of each gene were independently aligned using the MUSCLE algorithm under default parameters (http://www.ebi.ac.uk/Tools/msa/muscle/, [23]). After alignments, the matrix was checked by eye for any obvious misalignments and to detect potential cases of sequencing error due contamination or pseudogenes using Geneious and BioEdit v. 7.0.9.0 [24]. Nucleotide variation, substitution patterns, and genetic distances based on Kimura 2-parameters (K2P) were examined using MEGA v. 5.0 [25]. To evaluate the occurrence of substitution saturation in the sequences, the index of substitution saturation (Iss) described by Xia *et al*. [26] and Xia & Lemey [27] in DAMBE 5.3.48 [28] was estimated.

The genes *16S rRNA* and *COI* were concatenated into a single matrix for both phylogenetic inferences and divergence date estimates. Phylogenetic relationships among populations of *G*. *melanopleura* and between this species and outgroups were inferred by Bayesian inference (BI) and Maximum-likelihood (ML) methods. Sequences of *B*. *caudomaculatus*, the externalmost characiform in our dataset, were used to root the phylogenetic analyses. The best-fit nucleotide evolution model was estimated independently for each partition using MrModeltest v. 2.2 [29] based on the Akaike Information Criterion (AIC), in conjunction with PAUP* [30]. First, BI was conducted in MrBayes v. 3.2.6 [31]. Two independent Bayesian runs of 20 million generations with four chains of Markov chain Monte Carlo (MCMC) each were performed, saving trees each 500 generations. Chain convergence (Effective Sample Size–ESS values > 200) was checked using the likelihood plots for each run using Tracer v. 1.5.1 [32]. The Potential Scale Reduction Factor (PSRF) was also used to check chain convergence and burn-in; values close to one indicate good convergence between runs [33]. After a graphical analysis of the evolution of the likelihood scores, and checking for the stationarity of all model parameters, the first four thousand generations (10%) were discarded as burn-in. The remaining trees were used to calculate the consensus tree and posterior probability values were calculated to determine the level of support to the Bayesian topology. The ML phylogenetic reconstructions were performed using RAxML v. 8.0.24 [34], random starting trees, and a GTRGAMMA model of evolution. One thousand bootstrap pseudoreplicates were tested to investigate the support of each node in the most likely topology. In general, we interpreted bootstrap values above 75% in the ML analyses as well supported, and in the BI analyses, a posterior probability value of 0.99 was taken as a threshold. MrBayes and RAxML analyses were carried out at CIPRES Science Gateway portal [35].

Divergence time estimates were obtained by implementing a Bayesian relaxed clock model in the BEAST v. 1.7.2 [36] using the concatenated mitochondrial dataset in the CIPRES web portal and all clade-age inferences are presented as 95% highest posterior density (HPD). We used a relaxed clock with an uncorrelated lognormal distribution [37]; a starting tree was obtained from RAxML analysis; a macroevolutionary Birth–Death model for the diversification likelihood values; and under GTR+I+G model (as estimated in MrModeltest). We included two calibration points based on fossil records of the characids †*Paleotetra* (Eocene-Miocene, [38]) and †*Megacheirodon unicus* (Late Oligocene-Early Miocene, [39–40]). According to Mirande *et al*. [41], the genus †*Paleotetra* is included in a clade who is closely related to ((Aphyocharacinae (Aphyoditeinae, Cheirodontinae)), Stevardiinae). Thus, the first calibration point was implemented using a lognormal prior offset to 33.9 million years ago (Mya) with a standard deviation of 1 for the origin of the clade ((*C*. *ibicuhiensis*, *S*. *leptoura*), Stevardiinae) proposed by our ML starting tree. We used this estimated date based on the numerical age to Eocene-Oligocene (see [42]) horizon proposed to †*Paleotetra* by Weiss *et al*. [38]. The second calibration point was implemented using a lognormal prior offset to 27.5 Mya with a standard deviation of 1 for the origin of the subfamily Stevardiinae. This estimated date was based on the mean of the minimum age of 30–25 Mya proposed to †*M*. *unicus* [39–40], which was proposed as closely related to *Spintherobolus* by Bührnheim *et al*. [40]. We followed Forest [43] to choose the crown and stem groups. The analysis was performed in two independent runs with 100 million generations each, with parameters sampled every 10,000 steps, and a burn-in of 20%. We checked convergence between runs and analysis performance using Tracer, and accepted the results if ESS values were > 200. The resulting trees were combined in LogCombiner v. 1.7. 2 [36], the consensus species tree with the divergence times was obtained in the TreeAnnotator v. 1.7. 2 [36] and visualized in FigTree v. 1.3.1 [44].

The Generalized Mixed Yule-Coalescent (GMYC) method uses an ultrametric tree estimated from the sequences, aims to identify shifts in branching rate of the tree from a Yule (species) to coalescent (population) process [45,46]. We applied the GMYC method to evaluate if geographically isolated population samples of *G*. *melanopleura* represent independently evolving units. For this analysis, we also used the information of the mitochondrial concatenated dataset because the method treats them as a single locus. As the GMYC method requires a high number of species [47,48], in addition those used to phylogenetic inferences, more sequences deposited by Thomaz *et al*. [2] were obtained from the GenBank database (accession numbers: *16S* = KF209698-KF210029 and *COI* = KF210030-KF210276).

Because the GMYC requires an ultrametric tree, this was produced using BEAST under the following parameters: 150 million generations, with sampling every 25,000 generations; GTR+I+G model (as estimated in MrModeltest); Birth–Death prior; and lognormal relaxed molecular clock model. This model assumes that the rates of molecular evolution are uncorrelated but lognormally distributed among lineages [37]. A random tree was used as a starting tree for the MCMC searches and eight chains were run simultaneously. The above analysis was performed twice in the CIPRES portal. The distribution of log-likelihood scores was examined to determine the stationary phase for each search and to decide whether extra runs were required to achieve convergence using Tracer. All sampled topologies beneath the asymptote (15,000 generations) were discarded as part of a burn-in procedure, and the remaining trees were used to construct maximum clade credibility topology in TreeAnnotator. Lineage delimitation through the GMYC model was conducted using the standard parameters (interval = c(0, 10)) and a single threshold that specifies the transition time between to within species branching. Such analysis was conducted with the Species Limits by Threshold Statistics (“splits”) package (http://r-forge.r-project.org/projects/splits) using R v.3.0.0 [49] on standard parameters. The ‘gmyc’ function in R optimizes the likelihood function described by Pons *et al*. [45].

#### Phylogeographic analyses

Population structure tests, summary statistics, and demographic analyses were based on mitochondrial genes concatenated. We generated median-joining networks [50] using the program NETWORK v. 5.0.0.0 (www.fluxus-engineering.com) to study the relationships between haplotypes and their geographic distribution. Calculation of F-statistics (ΦST) and Analysis of Molecular Variance (AMOVA, [51]) were carried out in the program ARLEQUIN v. 3.5.2.2 [52]. These analyses were performed three times under the following criteria: (1) individuals of *G*. *melanopleura* sampled in the same basin were merged into a single population to quantify the amount of genetic structure amongst them (i.e., Tietê, Guaratuba, and Itanhaém); (2) to test if individuals sampled in upper Rio Tietê and coastal basins represented isolated populations, we considered specimens from Guaratuba and Itanhaém into a single population; and (3) we defined populations based on our phylogenetic analysis results, thus individuals from populations closely related were merged into a single. Summary statistics, such as nucleotide diversity per site (π), number of haplotypes (h), and haplotype diversity (Hd) were calculated in software DnaSP v. 5.10 [53]. To detect signals of demographic expansion, we applied the neutrality tests Fs. By Fu [54] and D, by Tajima [55] besides the population size change test R2 [56] in DnaSP. The significance of these tests was obtained based on 1,000 coalescent simulations.

### Morphological analyses

To evaluate if there are variations of morphological data within the range of *G*. *melanopleura* that would justify the recognition of more than one species among its geographically isolated populations, we analyzed features traditionally used to diagnose characids and glandulocaudines species (e.g., meristic and morphometric data, color pattern). Thus, counts and measurements follow Fink & Weitzman [57] and Menezes & Weitzman [58]. Measurements are given as percents of standard length (SL), except for subunits of the head given as percents of head length. Counts of supraneurals, branchiostegal rays, and vertebrae were taken from six cleared and stained (c&s) specimens, prepared following Taylor & Van Dyke [59]. Vertebrae of the Weberian apparatus were counted as four elements and the compound ural centrum as a single element. The data used for comparisons were taken from the original descriptions of *G*. *melanogenys* and *H*. *melanopleurus* (Eigenmann [9] and Ellis [12], respectively), as well as from Menezes & Weitzman [1] and from examination of topotypes and other specimens throughout the all known distribution of *G*. *melanopleura*. Comparative graphics of some meristic characters, represented by Tukey boxplot of ranked data (see [60]), was prepared from data of geographically isolated population samples of *G*. *melanopleura* plus *G*. *caerulea* with the program R v. 2.10.0 [61], available at http://www.r-project.org.

## Results

### Molecular approach

A total alignment of 1,043 base pairs (bp) was obtained for the mitochondrial genes *16S rRNA* (521 bp) and *COI* (522 bp). There were 145 and 213 variable sites for *16S* and *COI*, respectively. The coding sequences did not show insertions, deletions, stop-codons or sequencing errors due to contamination. The Iss index was significantly lower than the Iss.c (critical substitution saturation index), indicating no saturation in either transitions and transversions in both asymmetrical (Iss.cAsym) and symmetrical (Iss.cSym) topologies. The best-fit model of evolution estimated by MrModeltest for the all data matrices (*16S*, *COI*, and concatenated) was GTR+I+G.

**Fig 1:**
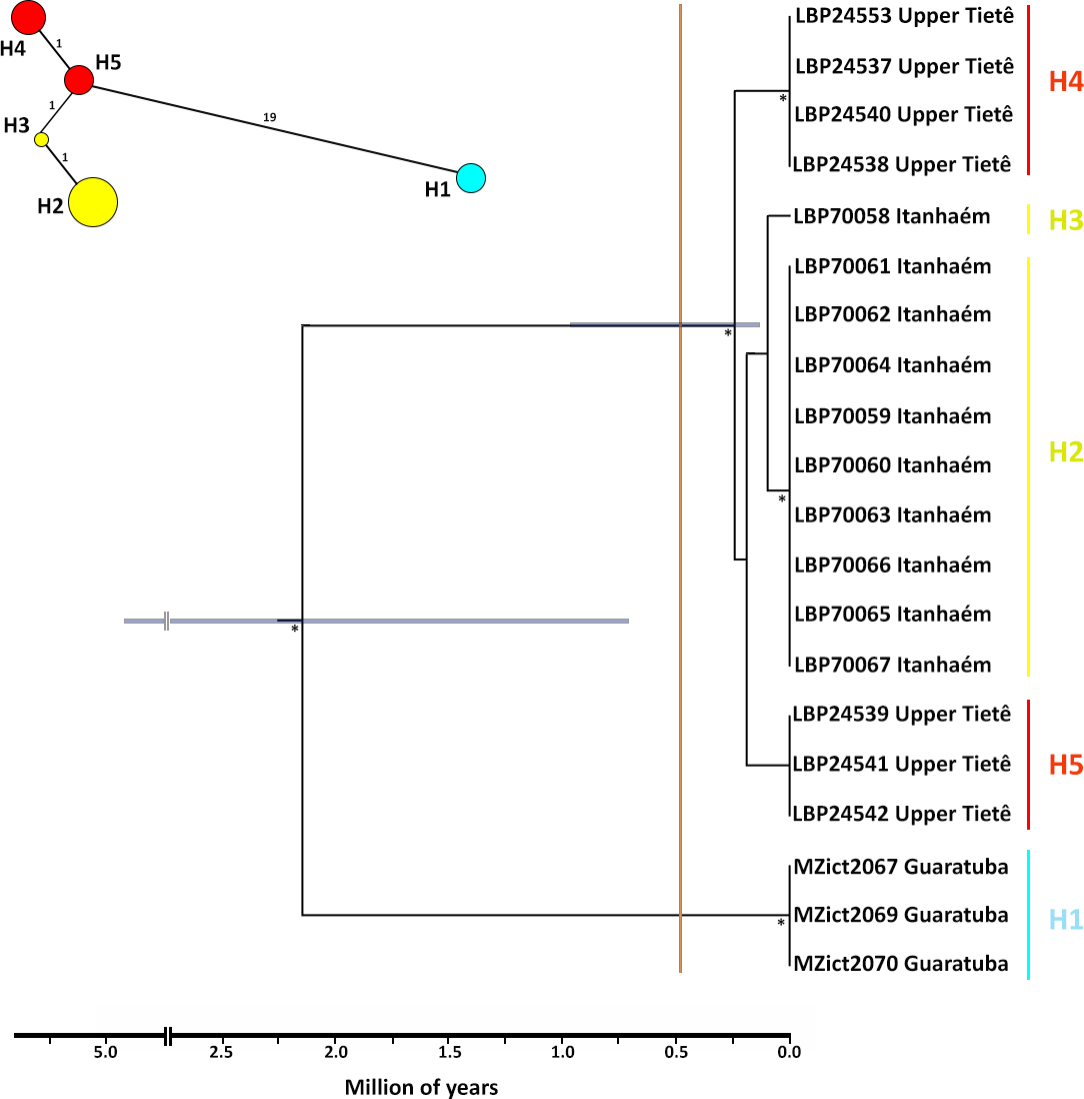
Relationships within *Glandulocauda melanopleura* inferred by the concatenated mtDNA dataset (*16S*+*COI*, 1,043 bp). Calibrated phylogenetic tree showing the delimitation of cladogenetic events at left of the vertical orange line obtained from GMYC analysis. Node bars indicate the threshold time for main cladogenesis. Asterisks indicates bootstrap and posterior probability values above 70% and 0.90, respectively. Haplotype network shows the genetic connectivity of the five haplotypes. Each circle represents a unique haplotype with its size proportional to haplotype frequency and numbers indicate mutational steps between haplotypes. Each color represents a population as in Fig 2.

Both phylogenetic methods (BI and ML) produced gene trees with very similar topologies in the outgroup and identical in *G*. *melanopleura*. In all phylogenetic analyses, *G*. *melanopleura* is recovered as monophyletic (S1 Fig), including two clearly distinguished and strongly supported clades (Fig 1): one is represented by specimens from Rio Guaratuba basin (GUA) that is the sister group of the clade formed by specimens from upper Tietê (UPT) and Itanhaém (ITA) river basins. The internal relationships in this clade, however, were not clearly resolved. According to the calibrated phylogeny, GUA diverged from the remaining populations of *G*. *melanopleura* in the Pleistocene, around 2.2 Mya (95%_HPD 0.7–4.7 Mya). The split of the populations from the UPT and ITA was more recent, in the late Pleistocene, almost Holocene, about 0.4 Mya (95%_HPD 0.1–0.9 Mya) (Fig 1).

Lineage delimitation analysis using the GMYC model estimated under a Birth-Death prior of branching rates showed a threshold time of -3.71x10^-3^. The maximum likelihood for the null model was 2067.178 and the maximum likelihood for the GMYC model was 2141.726. Using a single-threshold model from GMYC, the results are in agreement with the phylogenetic inferences and suggest the recognition of two lineages within *G*. *melanopleura* (Fig 1), being one distributed in both upper Tietê and Itanhaém river basins and the other in Rio Guaratuba drainage. Using the 2% standard Barcode threshold of genetic distance, calculated based on the *COI* matrix, we also identified these two lineages: the pairwise K2P value between population of the Rio Guaratuba and both populations of the Tietê and Itanhaém is 3% and null between the latter two.

The haplotype network was congruent with phylogenetic inferences and GMYC results and showed a strong genetic structure within *G*. *melanopleura* (Fig 1). In fact, this structuration was more evident because there was no shared haplotype among analyzed populations, indicating a clear association between genetic structure and geography concerning haplotypes. There is only one haplotype sampled in GUA (H1), two in each ITA (H2 and H3) and UPT (H4 and H5, with this being the central haplotype). As proposed by phylogenetic analyses, haplotypes from UPT are closely related to haplotypes sampled in ITA than those from GUA. The H5 (UPT) is separated by a single mutation step from the H3 (ITA), while 19 mutation steps separate it from H1 (GUA). In agreement with the haplotype networks, the AMOVA results showed high isolation among river basins for the mtDNA data, with the highest percentage of genetic variation observed among the localities when each river was analyzed separately (99.1%, p = 0.00). When we considered specimens from GUA and ITA as a single population, AMOVA results indicated that the highest percentage of genetic variation was observed within localities, 86.3% (p = 0.04), therefore the hypothetical group “coastal basins” (GUA+ITA) was not corroborated. On the other hand, when we considered specimens from UPT and ITA as a single population, the genetic structure within localities is low (1.6%, p = 0.00), corroborating the close relationship between UPT and ITA, proposed by both the phylogenetic analyses and haplotype network.

The summary statistics results are shown in Table 2. Overall, haplotype and nucleotide diversity were 0.747 and 0.00666, respectively. The highest values of Hd and π were found in UPT and these values were null in GUA, where there is a single haplotype. Neutrality (Fs and D) and population size change tests (R2) were not significant, thus no evidence of demographic expansion was detected by these tests.

**Table 2:** Summary statistics for mitochondrial genes (*16S* and *COI*) of *Glandulocauda melanopleura*.

Population	N	h	Hd (sd)	π (sd)	D	F_S_	R_2_
Upper Tietê	7	2	0.571 (0.119)	0.00055 (0.00012)	1.34164^ns^	0.856^ns^	0.2857^ns^
Itanhaém	10	2	0.200 (0.154)	0.00019 (0.00015)	-1.11173^ns^	-0.339^ns^	0.3000^ns^
Guaratuba	3	1	0.000	0.00000	-	-	-
TOTAL	20	5	0.747 (0.072)	0.00666 (0.00218)	0.17490^ns^	5.336^ns^	0.1474^ns^

N: sample size; h: number of haplotypes; Hd: haplotype diversity; sd: standard deviation; π: nucleotide diversity per site; D: Tajima’s test; Fs: Fu’s test; R_2_: Ramos-Onsins and Rozas’ test; ns: not-significant.

### Morphological approach

**Fig 2:**
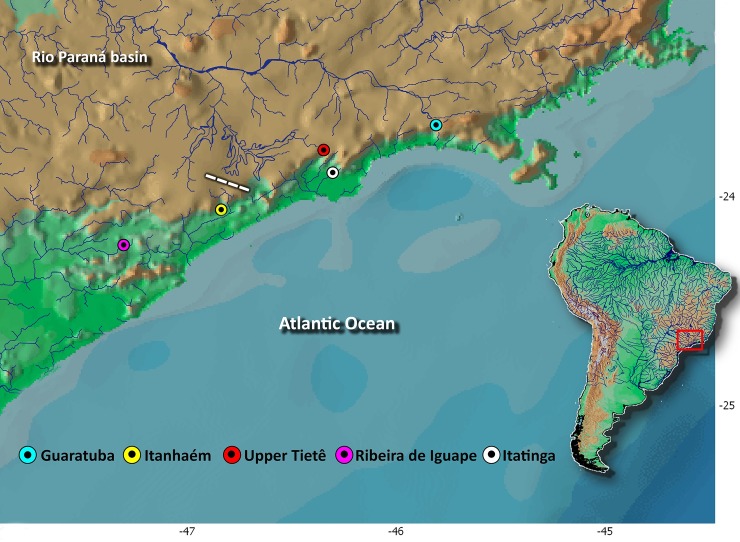
Map of southeastern Brazil showing geographical distribution of *Glandulocauda melanopleura*. Localities are upper Rio Tietê (Rio Paraná basin) and adjoining upper portions of the coastal rivers Guaratuba, Itatinga, Itanhaém, and Ribeira de Iguape flowing into the Atlantic Ocean in the state of São Paulo. Dashed line indicates the border area between upper Tietê and Itanhaém river basins, where headwater capture events probably occurred.

**Fig 3:**
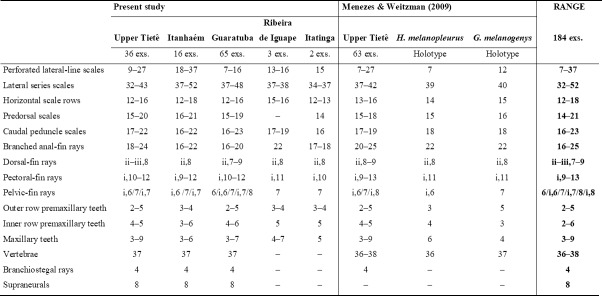
Meristics of *Glandulocauda melanopleura*. Are included data of holotype of *Hyphessobrycon melanopleurus*, holotype of *Glandulocauda melanogenys*, and additional data of specimens from Upper Tietê basin presented by Menezes & Weitzman [1], along with additional data of specimens from all known distribution of *G*. *melanopleura* obtained in this study. The range includes the holotypes of both species and –indicates unavailable data.

**Fig 4:**
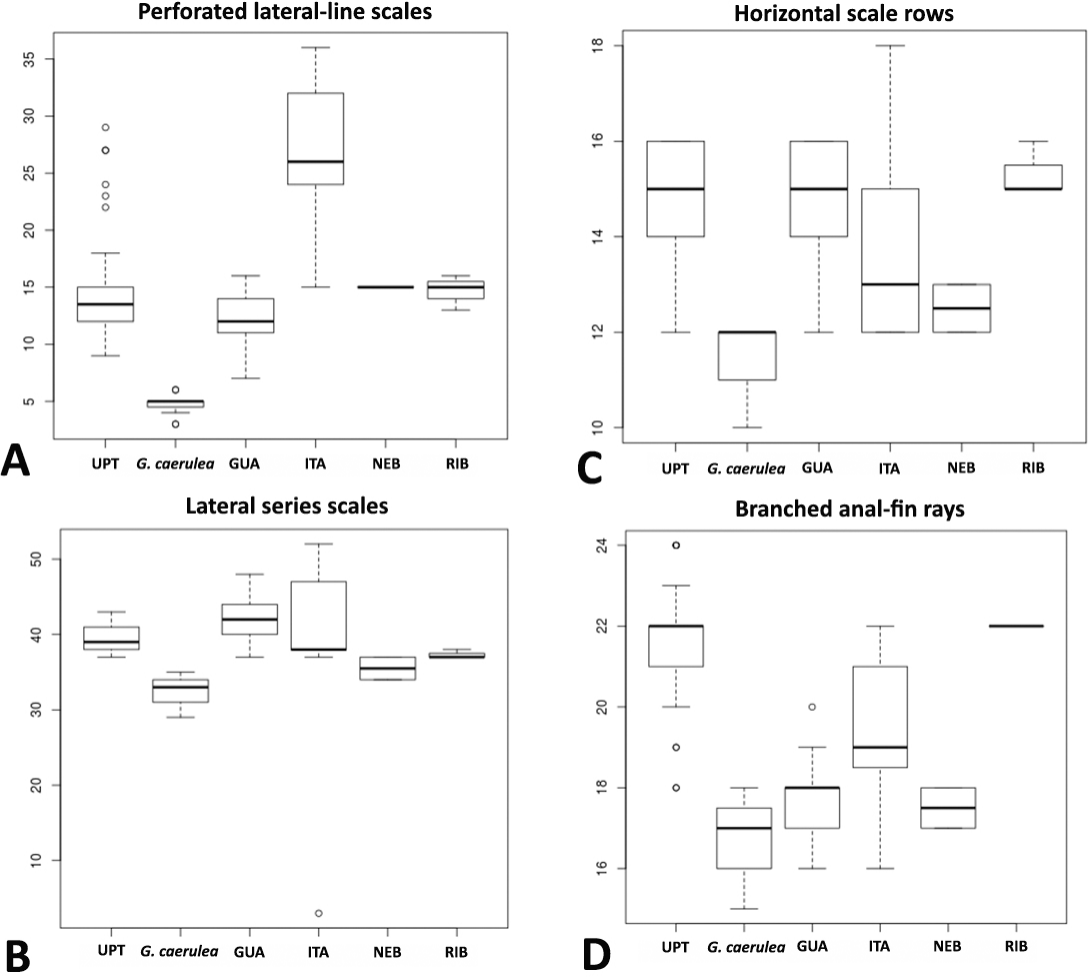
Comparative plots of samples of *Glandulocauda melanopleura* and *G*. *caerulea*. (A) number of perforated lateral-line scales, (B) number of lateral series scales, (C) number of horizontal scale rows on body, and (D) number of branched anal-fin rays. UPT: Upper Rio Tietê, GUA: Rio Guaratuba, ITA: Rio Itanhaém, NEB: Rio Itatinga, and RIB: Rio Ribeira de Iguape.

Morphological variation was evaluated among specimens of *G*. *melanopleura *from all known distribution (Fig 2), including those from Itatinga and Ribeira de Iguape river basins (S1 Appendix), which were not included in the molecular analyses due to lack of tissues. Meristic and morphometric data are presented in Fig 3 and S2 Table, respectively. Overall, a comparative analysis of these data showed broad overlap and absence of features supporting the recognition of more than one species within *G*. *melanopleura*. Also, the boxplots (Fig 4) revealed some intraspecific variation among geographically isolated populations, but with overlaps which led us to consider all samples conspecific.

**Fig 5:**
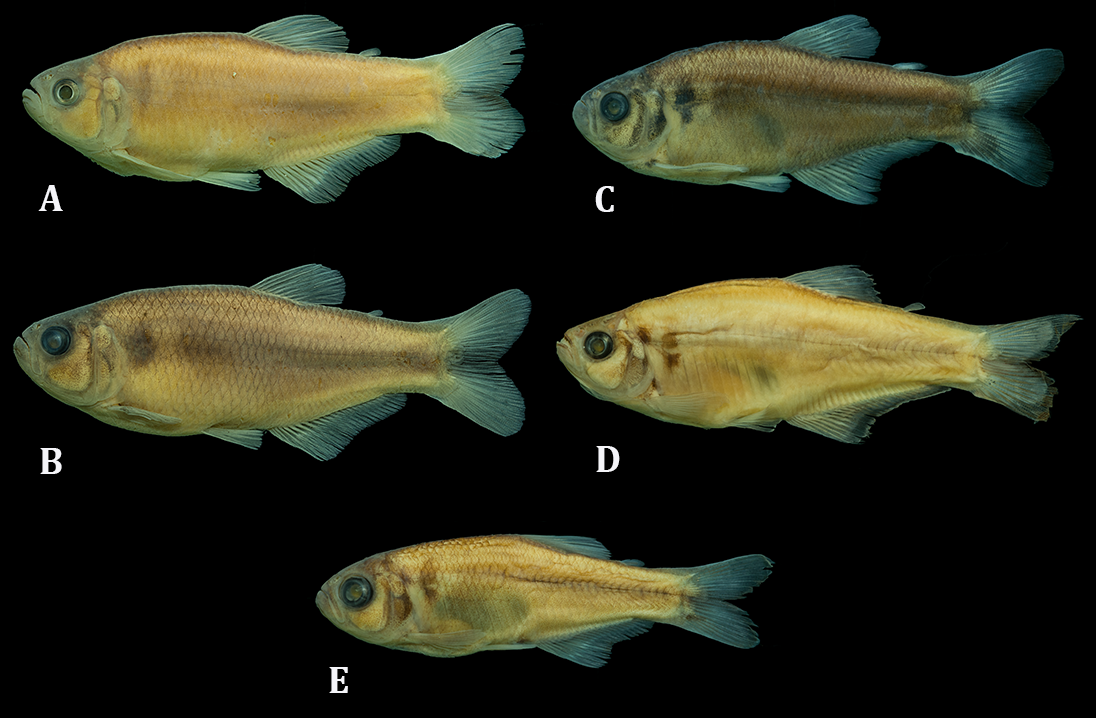
Color in alcohol of *Glandulocauda melanopleura* specimens from all known localities, Brazil, São Paulo state. (A) Upper Rio Tietê, MZUSP 86967, male, 58.4 mm SL, (B) Rio Itanhaém, MZUSP 111017, male, 50 mm SL, (C) Rio Guaratuba, MZUSP 115244, male, 39.4 mm SL, (D) Rio Ribeira de Iguape, MZUSP 79429, male, 48.9 mm SL, and (E) Rio Itatinga, DZSJRP 6613, juveline, 26.2 mm SL.

**Fig 6:**
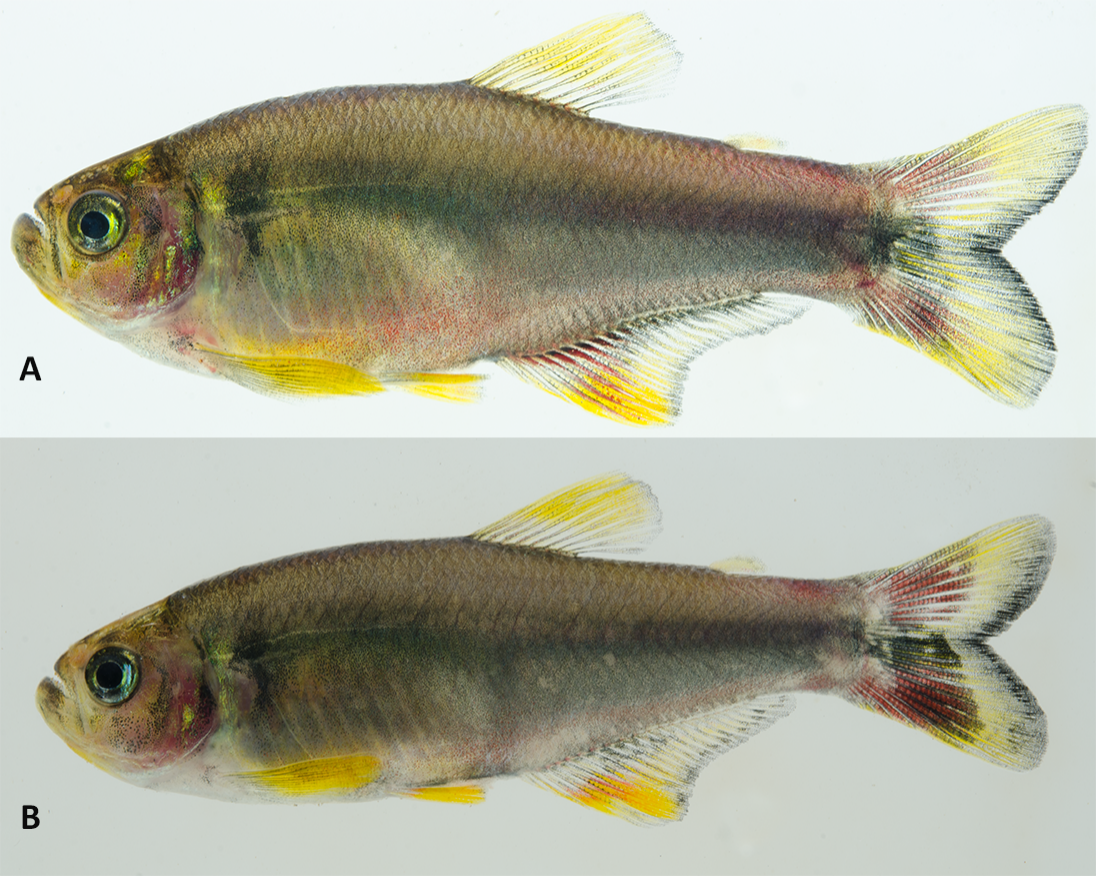
Color in life of *Glandulocauda melanopleura* from Rio Guaratuba basin, São Paulo state, Brazil. (A) male, MZUSP 115244, 39.4 mm SL, (B) female, MZUSP 115244, 35.0 mm SL.

**Fig 7:**
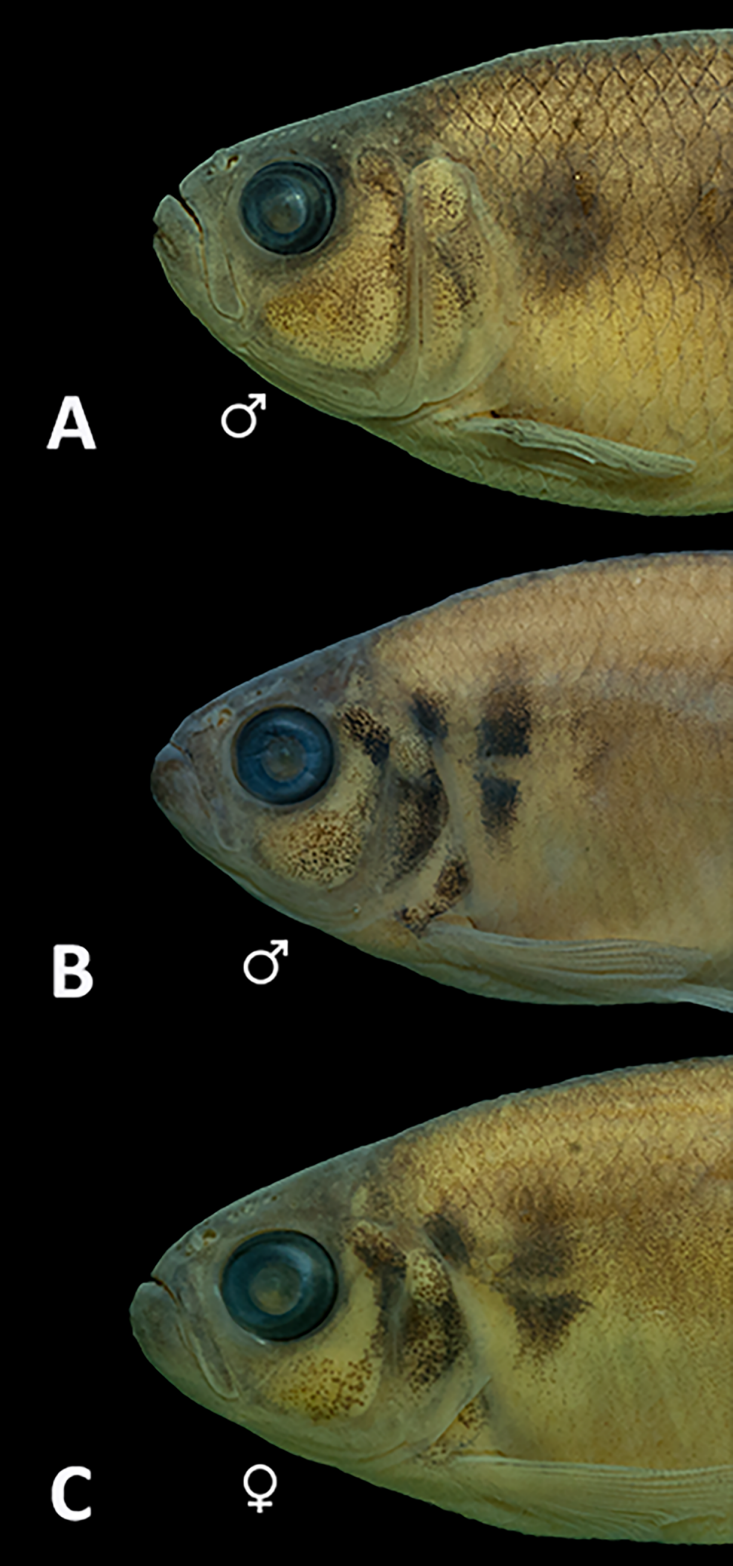
Detail of humeral and opecular regions of *Glanduulocauda melanopleura* showing the variation in the coloration pattern. Specimens from Rio Itanhaém basin, MZUSP 111017, (A) male, 55.8 mm SL, (B) male, 44.5 mm SL, and (C) female, 42.1 mm SL.

The color in alcohol of specimens from all localities is presented in Fig 5 and the live color pattern of individuals from Rio Guaratuba basin in Fig 6. A comparative analysis of both color patterns data obtained herein with those described by Menezes & Weitzman [1] also failed to demonstrate differences among geographically separated populations of *G*. *melanopleura*. Some specimens from the Rio Capivari presented a variation in their sexually dichromatic coloration pattern not expected for the species. According to Menezes & Weitzman ([1]: Figs 11 and 12), the vertically elongated humeral spot is more evident and darker in males than females, but we found both males with expected female coloration and females with the expected male coloration pattern within specimens from this basin (Fig 7). Additional comments on morphological characters and their variation are provided in the “Discussion”.

## Discussion

### Molecular approach

#### Phylogenetic inferences and GMYC analysis

Although *G*. *melanopleura* is a monophyletic entity, the results of all molecular analyses based on samples of topotypes plus specimens from Guaratuba and Itanhaém river basins indicated the presence of a strong population genetic structure within this species, with two clades–(UPT, ITA) and GUA–well supported. According to the GMYC, these clades represent different lineages which can be considered two putative species. This method estimates the transition point on a tree in a molecular clock hypothesis, before which all nodes reflect species diversification events and after which all nodes represent a population coalescent process [45] and has become a widely used method for delimiting species based on single-locus data [47,48,62–66]. Although the GMYC method has a strong theoretical basis, it typically generates a high number of operational taxonomic units (OTUs) [67,68], and this result has already been reported for some Neotropical freshwater fish species (e.g., [48,64,69]. Although the genetic distance be an useful evidence to detect and delimit species, it should is not the unique source of information used for this purpose [70–73]. Several authors have suggested that inferences regarding species boundaries based on genetic data alone are likely inadequate, and species delimitation should be conducted with consideration of other factors, such as morphology, geographical distribution, and behavior (e.g., [63,72,74–76]). According to Carstens *et al*. [72], incongruence across the results from different methods of species delimitation is relatively common and inferences drawn from these results should be conservative, thus in most contexts it is better to fail to delimit species than it is to falsely delimit entities. As we agree with these authors and no morphological diagnostic feature supporting the recognition of two species within *G*. *melanopleura* was found, we prefer to consider that specimens from all localities are conspecific and that genetic divergence observed may be result of geographical distance and not reflection of a species boundary. According to Avise [77], in species with low dispersal and gene flow, extinctions of intermediate haplotypes may contribute to the appearance of pronounced genetic gaps. Therefore, the genetic divergence found between specimens from UPT/ITA and GUA may be result of accumulated mutations over time, a relatively common scenario in species whose populations are geographically isolated [78], such as *G*. *melanopleura*.

#### Phylogeographic pattern and demography of *Glandulocauda melanopleura*

Haplotype networks and AMOVA also indicated presence of a strong genetic structure and allopatric fragmentation in *G*. *melanopleura*. These results are in agreement with what is expected in freshwater fish species [77,79], especially when the populations are distributed in drainage basins spatially disconnected such as *G*. *melanopleura*, as already reported by several authors for other species (e.g., [80–82]). The absence of shared haplotypes among the three analyzed basins indicated a genetic structuration higher than those proposed by phylogenetic and GMYC results, suggesting that these populations are separated for time enough to have reached reciprocal monophyly. Indeed, mitochondrial DNA tends to reach the reciprocal monophyly in a short time, especially among populations with low or no gene flow [83,84], such as *G*. *melanopleura*.

According to Avise [77] and Avise *et al*. [85], phylogeographic outcomes can be grouped into several categories that reflect different temporal scales and spatial aspects of population genealogical structure. Two of the five distinctive phylogeographic patterns proposed and discussed by these authors were identified within *G*. *melanopleura*. The split between (UPT, ITA) and GUA is a typical ‘Category I phylogeographic pattern’ (‘Deep gene tree, major lineages allopatric’), which is epitomized by the presence of spatially circumscribed haplogroups separated by relatively large mutational distances [77,85] and probably associated with the long-term extrinsic barriers to genetic exchange. According to Avise [77], this phylogeographic pattern is very common in freshwater fish species, which are usually distributed in isolated basins with gene flow restricted or absent, such as *G*. *melanopleura*. Another commonly encountered situation is one in which mtDNA parsimony networks are relatively continuous, with consistently small numbers of mutational steps between phylogenetically adjacent haplotypes, each of which is nonetheless confined to a subset of the geographic range of the species [85]. The phylogeographic pattern found between haplotypes from UPT and ITA can be categorized as such (‘Category III: Shallow gene tree, lineages allopatric’), in which most or all haplotypes are related closely, yet are localized geographically [77]. This pattern can be the result of a recent break of the gene flow [77,84], and this hypothesis was corroborated by our results of time divergence estimates.

Although the neutrality (Fs and D) and population size change tests (R2) failed to detect any evidence of demographic expansion, the combined analysis of traditional measures of haplotype and nucleotide diversity (Hd and π, respectively) provides an alternative means of inferring the general demographic history a population [77,86,87]. High Hd and low π (i.e., Hd > 0.5 and π < 0.5%), as found in UPT population (Hd = 0.6 and π = 0.05%), can be attributed to rapid population growth from an ancestral population with low effective size, since the rapid population growth enhances the retention of new mutations, but the time was yet short for an accumulation of large sequence differences [77,85,86]. Within the population from ITA, both Hd and π are small (0.2 and 0.02%, respectively) and it may represent a recent population bottleneck or founder event by single or a few mtDNA lineages [77,86]. Moreover, since larger values of π can be indicative of a larger and stable historical population size [77,87], probably ancestral populations of *G*. *melanopleura* inhabited the upper Rio Tietê basin or some paleodrainage of the Rio Paraná basin on upland areas of the Brazilian Crystalline Shield as proposed by Menezes *et al*. [3] and Ribeiro *et al*. [4].

#### Phylogeographic structure of *Glandulocauda melanopleura*, divergence time estimates, and headwater capture events

Menezes *et al*. [3] reviewed the biogeographic history of Glandulocaudini (at the time, Glandulocaudinae) based on phylogenetic and distributional data and proposed that the current allopatric distribution of *G*. *melanopleura* could be explained by the geomorphological history of the area where it occurs plus the environmental requirements of this species. Our results corroborate and complement the hypotheses and information provided by these authors.

*Glandulocauda melanopleura* is found only in first and second order clear water streams draining high altitudes ([1,3]. Although the species has been recorded for a blackwater stream (Rio Capivari basin) for the first time in this study, this locality has overall environmental characteristics very similar to those found in the other stretches where *G*. *melanopleura* inhabits (e.g., high altitude, cool flowing water, surrounded by dense rainforest vegetation). Thus, this new record does not refute the hypothesis that the ancestor of *G*. *melanopleura* probably presented a more widespread distribution along the upper Rio Paraná basin (as also suggested herein by the highest values of π found in the UPT population), but the mentioned environmental constraints of this species could be due to some local extinctions which resulted in its relict disjoint distribution [3].

Since freshwater fishes have limited capacity to disperse across marine or terrestrial barriers, being physiologically confined to rivers and streams after their formation [88–90], the distribution of species/populations across multiple basins may be explained by river captures or dispersal associated with temporary connections [90–92]. Paleoconnections due to sea level retreat played a significant role in the diversification and recent structuration of the ichthyofauna in lowland along the Brazilian coastal drainages [91,92], while events of a relatively continuous history of headwater stream capture can explain patterns of drainage isolation and coalescence across watershed divides, mainly among basins draining the Brazilian shield [4,8,90,93]. Since *G*. *melanopleura* is a typical upland species, restricted to streams draining the Brazilian crystalline shield, events of headwater capture can be considered an important causal explanation for the current pattern of allopatric distribution of this species throughout the distinct basins of southeastern Brazil [3,5]. A fluvial capture (i.e., river or stream piracy) corresponds to the natural divert of part or all river discharge from its own bed to a neighboring drainage system, promoting the expansion of a basin in detriment of the other [94]. It is a geomorphological phenomenon resulting of differential rates of erosion or tectonic uplift, or from damming by a landslide or ice sheet [93,95]. In most cases the capture involves both geodispersal and vicariance events [93,96] and has been used as main explanation for the pattern distribution of several freshwater fish species (e.g., [4,5,6,96,97]). Thus, this process can allow both faunal interchange and formation of isolated populations and our results indicates that both occurred in *G*. *melanopleura* and shaped the pattern of distribution of the species and its haplotypes, as discussed in detail below.

Our analysis suggests that the ancestor of *G*. *melanopleura* originated in the upper Rio Paraná basin 7.8 Mya (95%_HPD 3.6–13.1 Mya) (estimated age to split of *L*. *weitzmani* and *G*. *melanopleura*). Therefore, after that at least two headwater capture events must have occurred to explain the occurrence of this species in upper portions of the coastal rivers Guaratuba and Itanhaém. Among the several headwater stream piracy events proposed and/or documented in basins draining the eastern part of Brazil, mainly in the region of the Brazilian crystalline shield (e.g., [3,4,6,7,98–100]), the “Rio Guaratuba capture” probably is one of the most discussed (e.g., [5,6,101–104]). Ribeiro *et al*. [5] studied in detail the ichthyofauna of the Atlantic Rainforest of Estação Biológica de Boraceia and proposed that the upper portion of Rio Guaratuba was captured and diverted away from its original course in the direction of the Paraná river basin to become a coastal river. These authors presented several ichthyological (including distribution data of *G*. *melanopleura*, at that time *G*. *melanogenys*) and geological evidences to corroborate their hypothesis that the fish fauna of the upper Guaratuba is an evident testimony of the tectonic process that allowed faunal interchange between isolated river basins and these evidences will not be repeated here. Later, some authors (e.g., [102,104]) presented and discussed more geological evidences, explaining in detail how this capture should have occurred. According to Oliveira [102] and Oliveira & Neto [104], the piracy of the Rio Guaratuba is as typical river capture triggered by “headward erosion”. This type of capture occurs when two adjacent rivers are located at distinct altitudes and the tributaries draining the lower course cause regressive erosion of their headwaters, crossing the interflow and capturing the watercourse located at the highest-level high [102]. The capture of the Rio Guaratuba was the result of the regressive erosion of the Serra do Mar escarpment at Bertioga, São Paulo [5,102]. The age of the river piracy was inferred to be of Late Pleistocene-Holocene at the last phases of tectonic reactivations of the Continental Rift of Southeastern Brazil (CRSB, [5,103,105]). Based on our results of divergence time estimates, we added new support to this hypothesis, corroborated the young geologic age of this event (around 2.2 Mya). According to Ribeiro *et al*. [5], the fact that the fish species occurring in the upper Rio Guaratuba are identical to the ones that occur in the upper Rio Tietê indicates that relatively little time has elapsed since this vicariant event took place. However, our molecular results indicate that, although it is a recent event, the time elapsed since the separation of these basins was sufficient for the formation of structured populations of *G*. *melanopleura* in both basins, which represent distinct lineages with endemic haplotypes.

The events of capture in upland rivers, which became Atlantic tributaries, occurred several times and continuously, especially in the Brazilian southeastern continental margin, leading to the mixed distributional patterns between Atlantic tributaries and the upland crystalline shield areas [4,5]. As well as Ribeiro *et al*. [5], we also combined biological and geological data and proposed another headwater stream capture event that shaped the distribution pattern of *G*. *melanopleura*, and this piracy occurred between the Rio Capivari and upper Tietê basins. The Rio Capivari drainage is located at a well preserved environmental Protection area called “APA Capivari-Monos/Núcleo Curucutu”, which is included within the limits of the Parque Estadual da Serra do Mar, a conservation unit which encompasses most of the Serra do Mar range in the São Paulo state. As in the Estação Biológica de Boraceia, this area also acts as a divide of diametrically opposing drainage systems: the Rio Embu-Guaçu, which drains to the upper Rio Tietê basin, and three tributaries of the upper Rio Itanhaém, an isolated basin draining directly into the Atlantic Ocean. Among these tributaries is the Rio Capivari, an affluent of the Rio Branco that presents a very complex geomorphological layout [106,107], probably resulting of the piracy event. The Rio Capivari has its upper and lower courses drastically separated by an escarpment of about 700 m height, consisting in the precipitous front of the Serra do Mar coastal range. It flows south-north direction toward the Rio Tietê from its springs, almost parallel to the Rio Embu-Guaçu, thus converges abruptly 130° to west, flows west-east until joining the Rio Monos, converging towards south through the escarpment of the Serra do Mar until draining into the Rio Branco, at Itanhaém city, São Paulo [106,107]. The abrupt turn in the course of the Rio Capivari next to the watershed with the Rio Embu-Guaçu can be attributable to stream piracy and this evidence is known as “elbow of capture” (see course of this river in the Fig 3). Besides this, Ab'Saber [107] provided other geomorphological evidences that the Rio Capivari was captured and diverted away from its original course in the direction of the Rio Paraná basin to become a coastal basin tributary. As well as Ribeiro *et al*. [5], we also propose that the fish fauna of Rio Capivari and upper Rio Tietê is an evident testimony of this capture. Besides *G*. *melanopleura*, several other freshwater fish species, which have a recognized restricted distribution pattern and are not found in Brazilian coastal drainages, are shared between upper Capivari and Tietê basins, such as *Astyanax bockmani* (e.g., MZUSP 108591; MZUSP 108649), *Atlantirivulus santensis* (e.g., MZUSP 108627; MZUSP 108647), *Pseudotocinclus tietensis* (e.g., MZUSP 108578; MZUSP 108642), and *Trichomycterus paolence* (e.g., MZUSP 108622; MZUSP 108930). According to our results of divergence time estimates presented herein, this capture event occurred very recently, probably in the late Pleistocene almost Holocene (about 0.4 Mya). Furthermore, these results corroborate the hypothesis of Ab'Saber [107] that the Rio Capivari was still an affluent of the upper Tietê in the Pliocene and part of Quaternary. The location of the capture plus its relatively young geologic age takes us to propose that it was also the result of the last phases of tectonic reactivations of the CRSB. As previously mentioned, our results indicate that in spite of being a very recent event that failed to give rise to two distinct lineages, the time elapsed since the disruption of gene flow was sufficient for establishment of endemic haplotypes in these basins.

According to Menezes *et al*. [3], the upper portions of both the Itatinga and the Ribeira de Iguape river basins are captured stretches that primitively drained to the Paraná basin before the tectonic reactivation of the CRSB that caused drainage rearrangements in the area. Although the geomorphologic history of the area may require further geological studies, the relatively recent (Quaternary) connection between the upper Rio Itatinga and the nearby Tietê headwaters was also proposed by other authors (e.g., [6,14]). Therefore, the shared presence of *G*. *melanopleura* in headwaters of these basins should also be a consequence of river captures. However, since we did not have access to tissue samples of specimens from these drainages and there are no available molecular data, we can neither corroborate these hypotheses nor estimate the date of captures.

### Morphological variation among allopatric populations of *Glandulocauda melanopleura*

Most specimens of *G*. *melanopleura* examined by Menezes & Weitzman [1] in their review of the systematics of the Glandulocaudini (at that time, Glandulocaudinae) is from the upper Tietê (type locality and near it), but they also examined three individuals from the Ribeira de Iguape (MZUSP 79429) and 31 from the Rio Guaratuba basin (MZUSP 48511 and 84412). In fact, the locality data of MZUSP 48511 provided by these authors was “córrego Mutuca, Estação Biológica de Boraceia” and in this ecological reserve there are two homonymous streams, known as “córrego Mutuca”: one is a tributary of the Rio Guaratuba and the other of the Rio Claro, an affluent of the upper Tietê basin. We plotted the coordinate data on the map and there is no doubt that these specimens are from the upper Guaratuba basin.

The comparative analysis of the morphometric and meristic data compiled and provided by Menezes & Weitzman [1] with data of specimens from the wide distribution of *G*. *melanopleura* obtained herein revealed some variation of morphological characters among geographically separated populations. According to these authors, the specimens from the upper Rio Guaratuba basin and four of 59 examined individuals from upper Tietê basin have a reduced number of branched anal-fin rays (17–20) when compared with most of the specimens from the type locality or near it (20–25), and these values are in the range of those for *G*. *caerulea* (17–19). However, since these specimens are very similar to *G*. *melanopleura* with respect to all the other meristic and morphometric characters, the authors did not include them in their data set and suggested that a detailed analysis of more individuals from these locations was necessary. This analysis was carried on herein. Indeed, our results indicated that most examined specimens from the upper Tietê basin (31 of 36 individuals) have high values of branched anal-fin rays (20 or more) and the same occurs with specimens from the Ribeira de Iguape (22). However, although populations from the upper Guaratuba and Itatinga basins present a clear tendency to have lower number of branched anal-fin rays (16–19, only one of 66 analyzed specimens has 20), all range of variation was observed among specimens from the upper Rio Itanhaém drainage (16–22), indicating the plasticity of this character within *G*. *melanopleura*. Moreover, if we considered populations from the upper Tietê and Itanhaém as a single lineage as suggested by the GMYC and phylogenetic analyses, all values found in specimens from the Guaratuba basin are in the range of those for the lineage (UPT, ITA) (16–20 and 16–25, respectively). Besides the number of branched anal-fin rays, Menezes & Weitzman (2009: 315) also highlighted that 10 specimens from the Guaratuba basin (MZUSP 84412) present counts of longitudinal scale rows from dorsal-fin origin to anal-fin origin and scales around the caudal peduncle with higher values than those found in specimens from the type locality or near it (16–17, mean 16.6 *vs* 13–16, mean 15.1 and 18–20, mean 19.3 *vs*. 17–19, mean 18.0, respectively). As suggested by these authors, we analyzed in this study more specimens from these locations, mainly from the Rio Guaratuba, and our results indicated a complete overlap in the range of both features, with a little increase in these ranges for the species (see Fig 3).

As in all glandulocaudin species, *G*. *melanopleura* has the lateral line incomplete [1,9], which means that there are pored plus not pored scales covering the literal line channel. On the other hand, unlike all species of the tribe, *G*. *melanopleura* present a high range of variation in the number of perforated scales on the lateral line series (7–27 *vs*. 4–8 in *G*. *caerulea*, 1–7 in *L*. *weitzmani*, and up to 5–10 among *Mimagoniates* species, Menezes & Weitzman 2009). Within the population from the Rio Capivari basin, analyzed in this study for the first time, the lateral-line perforation is even more variable. Of the 19 analyzed specimens (MZUSP 106577, 108724, 108621, and 111017), five have more than 27 pored scales, three have a discontinuous lateral-line pattern (i.e., pored scales interspersed with non-pored ones), and one specimen has a completely pored lateral line, a condition never mentioned before for the species. Although the presence of a variable lateral-line perforation within a species is an unusual or poorly documented condition among the species of Characidae, it was described for other small characids genera, including *Hemigrammus* (e.g., *H*. *ataktos*; *H*. *barrigonae*), *Moenkhausia* (e.g., *M*. *celibela*; *M*. *cotinho*), and *Odontostilbe* (e.g., *O*. *dialeptura*) [108]. According to Fink & Weitzman [57], *O*. *dialeptura* tends to have an incomplete lateral line, but some population samples usually have a complete or almost complete lateral line, such as occurs in *G*. *melanopleura*. Also, including data from the Itanhaém specimens, the range of variation of scales on the lateral line of the species increased from 37–42 to 32–52.

Although *G*. *melanopleura* is slightly more widespread as previously proposed (e.g., [13,109]), it is endemic to the crystalline shield of southeastern Brazil in high altitudes areas (around 700–850 m a.s.l), including headwaters of both Tietê and coastal basins. Here, it is worth pointing out that several expeditions to the middle and lower portions of these basins were carried out (including in this study), and these collecting efforts did not reveal additional distributional records of *G*. *melanopleura*. According to some authors (e.g., [73,110]), the morphological variation found among populations of freshwater fish species within small drainages can be due to elevation. As the basins where *G*. *melanopleura* was sampled draining areas with similar altitudes, we concluded that this cause and effect relationship can not be established for this species. Morphological variation among populations of *G*. *melanopleura* is not clinal and the phenotypic diversity appears to be independent of genetic proximity among them. Zamudio *et al*. [111] obtained similar results for *Trichogenes longipinnis*, a freshwater fish species narrowly distributed and endemic to small streams draining the Atlantic Forest of SE Brazil, such as *G*. *melanopleura*. As discussed in detail by these authors [111], the fixation of divergent morphological patterns in specimens of *T*. *longipinnis* from the same drainage can be due to stochastic events during the founding of populations or historical reductions in population sizes. We agree with their hypothesis and also associate the comparatively high degree of phenotypic variation of some features among specimens from the Rio Itanhaém basin to genetic drift and fixation of different patterns of these features in the Rio Capivari.

According to Menezes & Weitzman [1], *G*. *melanopleura* differs from *G*. *caerulea*, its unique congener, by having 20–24 branched anal-fin rays (*vs*. 15–18), 13–16 horizontal scale rows from dorsal-fin origin to anal-fin origin (*vs*. 11–13), and 37–42 lateral series scales (*vs*. 31–35). These features were also presented in the original description as being diagnostic ([9]: 168). Although the analysis of additional material (more specimens and populations) carried out in this study had indicated a slight overlap of these features, these species can be easily distinguished by the lower jaw protruding, extending slightly anterior to the upper jaw in *G*. *melanopleura* (*vs*. lower jaw equal to or slightly shorter than upper jaw, see S2A Fig), caudal-fin squamation pattern of mature males (see [1]: Figs 15 and 24, respectively), and live color pattern of males (overall brown- to yellowish in *G*. *melanopleura vs*. mainly bluish in *G*. *caerulea* (see S2B Fig).

### Conservation status of *Glandulocauda melanopleura*

Although all populations of *G*. *melanopleura* are considered as belonging to the same species and no taxonomic change has been proposed herein, our study has a practical implication for its conservation due the existence of different lineages and the presence of endemic haplotypes. Almost 10 years ago, the conservation status of *G*. *melanopleura* was classified as Vulnerable (VU, i.e., highly endangered in the wild) according to the International Union for Conservation of Nature (IUCN) categories and criteria [112] (see [113]). However, the ‘IUCN Red List of Threatened Species’ was updated (MMA 45/2014, available at http://www.icmbio.gov.br/cepsul/images/stories/legislacao/Portaria/2014/p_mma_445_2014_lista_peixes_amea%C3%A7ados_extin%C3%A7%C3%A3o.pdf) and, nowadays, this species is not included in any threatened category, probably due to the new records of distribution and its occurrence nearby ecological reserves or stations (e.g., REBIO do Alto da Serra de Paranapiacaba, Estação Biológica de Boraceia, Parque das Neblinas, and APA Capivari-Monos). However, our results indicate that this change must be re-evaluated. Even though its distribution is broader than previously thought, in each river basin where *G*. *melanopleura* occurs, there are endemic haplotypes, which must be preserved, since the genetic diversity is crucial to ensure the survival of species [111,114]. Moreover, its distribution is still very restricted and some local extinction may already have happened. Three specimens from the Rio Ribeira de Iguape were sampled in 1999 (MZUSP 79429), downstream a dam inside a farm, and after that there is not any additional record, even with several expeditions done with this aim in the last years, including during this study. Thus, this species may have disappeared in the Ribeira de Iguape basin due to pollution and farmer activities and/or damming. *Glandulocauda melanopleura* apparently requires cool flowing waters and forested areas for successful reproduction and survival [1,115] which are becoming increasingly rare. Menezes & Weitzman ([1]: 316) listed several human activities that could be affecting the survival of *G*. *melanopleura* at that time, but we believe that these threatening activities still prevail. Therefore, we not only think that *G*. *melanopleura* deserves special attention from the conservation viewpoint, but also that any conservation measure should take into consideration genetic units of the species. According to Peñas *et al*. [114], haplotypes endemic to restricted areas (such as *G*. *melanopleura*) represent singular genetic variants that may have evolved separately from each other and, therefore, they deserve particular conservation effort.

## Supporting information

S1 Appendix Material examined of *Glandulocauda melanopleura* and *G*. *caerulea* in the morphological analyses. (DOCX)

S1 Fig Abbreviated Maximum Likelihood (ML) of *Glandulocauda melanopleura* tree based on mitochondrial concatenated dataset (1,043 bp). In highlight, the placement of the species within Glandulocaudini and relationships among three populations of this species: upper Rio Tietê (UPT), Rio Itanhaém (ITA), and Rio Guaratuba (GUA). Numbers at branches are bootstrap values from 1,000 bootstrap pseudoreplicates obtained from ML analysis and posterior probabilities obtained in the Bayesian Inference analysis. Values below 70% and 0.90 (–) are not shown.
(TIF)

S2 Fig Representatives of *Glandulocauda caerulea*. All from upper Rio Iguaçu basin, Paraná state, Brazil. (A) fixed specimen, MZUSP 97663, male, 40.8 mm SL and (B) alive specimen, MZUSP 117479, male, 34.1 mm SL.
(TIF)

S1 File *16S rRNA* sequences produced in this study. (TXT)

S2 File *COI* sequences produced in this study. (TXT)

S1 Table List of lot number, voucher, and GenBank accession number for each taxon used in this study (with exception of *Glandulocauda melanopleura*). (DOCX)

S2 Table Morphometrics of *Glandulocauda melanopleura*. (XLS)

## References

[1] Menezes NA, Weitzman SH. Systematics of the Neotropical fish subfamily Glandulocaudinae (Teleostei: Characiformes: Characidae). Neotrop Ichthyol. 2009;7: 295–370.

[2] Thomaz AT, Arcila D, Ortí G, Malabarba LR. Molecular phylogeny of the subfamily Stevardiinae Gill, 1858 (Characiformes: Characidae): classification and the evolution of reproductive traits. BMC Evol Biol. 2015;15: 1–25. 26195030 10.1186/s12862-015-0403-4 PMC4509481

[3] Menezes NA, Ribeiro AC, Weitzman SH, Torres RA. Biogeography of the Glandulocaudinae (Teleostei: Characiformes: Characidae) revisited: phylogenetic patterns, historical geology and genetic connectivity. Zootaxa. 2008;1726: 33–48.

[4] Ribeiro AC. Tectonic history and the biogeography of the freshwater fishes from the coastal drainages of eastern Brazil: an example of faunal evolution associated with a divergent continental margin. Neotrop Ichthyol. 2006;4: 225–246.

[5] Ribeiro AC, Lima FCT, Ricomini C, Menezes NA. Fishes of the Atlantic Rainforest of Boracéia: testimony of the Quaternary fault reactivation within a Neoproterozoic tectonic province in Southeastern Brazil. Ichthyol. Explor. Freshw. 2006:17: 625–630.

[6] Buckup PA. The Eastern Brazilian Shield In: Albert JS, Reis RE, editors, Historical Biogeography of Neotropical Freshwater Fishes. California: University of California Press; 2011 pp. 203–210.

[7] Lima FCT, Ribeiro AC. Continental-Scale Tectonic Controls of Biogeography and Ecology In: Albert JS, Reis RE, editors, Historical Biogeography of Neotropical Freshwater Fishes. California: University of California Press; 2011 pp. 145–184.

[8] Camelier P, Zanata AM. Biogeography of freshwater fishes from the Northeastern Mata Atlântica freshwater ecoregion: distribution, endemism, and area relationships. Neotrop Ichthyol. 2014;12: 683–698.

[9] Eigenmann CH. III. New characins in the collections of the Carnegie Museum. Ann Carnegie Mus. 1911;8: 164–181.

[10] Géry J. *Glandulocauda terofali* sp. nov., un nouveau Poisson characoïde de la République Argentine, avec une note sur la “glande” caudale des Stevardiidi. Opusc Zool. 1964;78: 1–12.

[11] Weitzman SH, Fink SV. Xenurobryconin phylogeny and putative pheromone pumps in glandulocaudine fishes (Teleostei: Characidae). Smithson Contrib Zool. 1985;421: 1–121.

[12] Ellis MD. 1911. On the species of *Hasemania*, *Hyphessobrycon*, and *Hemigrammus* collected by J. D. Haseman for the Carnegie Museum. Ann Carnegie Mus. 1911;8: 148–163.

[13] Weitzman SH. 2003. Subfamily Glandulocaudinae (Characins, tetras) In: Reis RE, Kullander SO, Ferraris CJ Jr., editors. Check list of the freshwater fishes of South and Central America. Porto Alegre: Edipucrs; 2003 pp. 222–230.

[14] Serra JP, Carvalho FR, Langeani F. Ichthyofauna of the rio Itatinga in the Parque das Neblinas, Bertioga, São Paulo State: composition and biogeography. Biota Neotropica. 2007;7: 81–86.

[15] Myers N, Mittermeier RA, Mittermeier CG, Fonseca GAB, Kent J. Biodiversity hotspots for conservation priorities. Nature. 2000;403: 853–858. doi: 10.1038/35002501 10706275 10.1038/35002501

[16] Lima RAF, Mori DP, Pitta G, Melito MO, Bello C, Magnago LF, et al How much do we know about the endangered Atlantic Forest? Reviewing nearly 70 years of information on tree community surveys. Biodivers Conserv. 2015;24: 2135–2148.

[17] Oliveira C, Avelino GS, Abe KT, Mariguela TC, Benine RC, Ortí G, et al Phylogenetic relationships within the speciose family Characidae (Teleostei: Ostariophysi: Characiformes) based on multilocus analysis and extensive ingroup sampling. BMC Evol Biol. 2011;11: 275 doi: 10.1186/1471-2148-11-275 21943181 10.1186/1471-2148-11-275 PMC3190395

[18] Pereira LHG, Hanner R, Foresti F, Oliveira C. Can DNA barcoding accurately discriminate megadiverse Neotropical freshwater fish fauna? BMC Genet. 2013;14: 1–14. 23497346 10.1186/1471-2156-14-20 PMC3608943

[19] Fricke R, Eschmeyer WN. Guide to fish collections. 2017. Available from http://researcharchive.calacademy.org/research/ichthyology/catalog/collections.asp

[20] Palumbi SR. Nucleic acids II: the polymerase chain reaction In: Hillis D, Moritz C, Mable B, editors. Molecular Systematics. Sunderland: Sinauer Associates Inc. 1996 pp. 205–247.

[21] Ward RD, Zemlak TS, Innes BH, Last PR, Hebert PND. DNA barcoding Australia’s fish species. Philos Trans R Soc Lond B Biol Sci. 2005;360: 1847–1857. doi: 10.1098/rstb.2005.1716 16214743 10.1098/rstb.2005.1716 PMC1609232

[22] Kearse M, Moir R, Wilson A, Stones-Havas S, Cheung M, Sturrock S, et al Geneious Basic: an integrated and extendable desktop software platform for the organization and analysis of sequence data. Bioinformatics. 2012;28: 1647–1649. doi: 10.1093/bioinformatics/bts199 22543367 10.1093/bioinformatics/bts199 PMC3371832

[23] Edgar R. Muscle: a multiple sequence alignment method with reduced time and space complexity. BMC Bioinformatics. 2004;5: 1–19. 15318951 10.1186/1471-2105-5-113 PMC517706

[24] Hall TA. BioEdit: a user-friendly biological sequence alignment editor and analysis program for Windows 95/98/NT. Nucleic Acids Symp Ser (Oxf). 1999;41: 95–98.

[25] Tamura K, Peterson D, Peterson N, Stecher G, Nei M, Kumar S. MEGA5: Molecular Evolutionary Genetics Analysis Using Maximum Likelihood, Evolutionary Distance, and Maximum Parsimony Methods. Mol Biol Evol. 2011;28: 2731–2739. doi: 10.1093/molbev/msr121 21546353 10.1093/molbev/msr121 PMC3203626

[26] Xia X, Xie Z, Salemi M, Chen L, Wang Y. An index of substitution saturation and its application. Mol Phylogenet Evol. 2003;26: 1–7. 12470932 10.1016/s1055-7903(02)00326-3

[27] Xia X, Lemey P. Assessing substitution saturation with DAMBE In: Lemey P, Salemi M, Vandamme AM, editors. The Phylogenetic Handbook: A Practical Approach to DNA and Protein Phylogeny. Cambridge: University Press 2009 pp. 615–630.

[28] Xia X. DAMBE5: A comprehensive software package for data analysis in molecular biology and evolution. Mol Biol Evol. 2013;30: 1720–1728. doi: 10.1093/molbev/mst064 23564938 10.1093/molbev/mst064 PMC3684854

[29] Nylander JAA. MrModeltest v2. Program distributed by the author Evolutionary Biology Centre, Uppsala University 2014

[30] Swofford DL. PAUP*. Phylogenetic Analysis Using Parsimony (*and Other Methods). Version 4.0. Sinauer Associates, Sunderland, Massachusetts 1998.

[31] Ronquist F, Teslenko M, van der Mark P, Ayres DL, Darling A, Höhna S, et al MrBayes 3.2: Efficient Bayesian Phylogenetic Inference and Model Choice Across a Large Model Space. Syst Biol. 2012;61: 539–542. doi: 10.1093/sysbio/sys029 22357727 10.1093/sysbio/sys029 PMC3329765

[32] Rambaut A, Drummond AJ. Tracer, version 1.5. 2009.

[33] Gelman A, Donal BR. Inference from iterative simulation using multiple sequences. Stat Sci. 1992; 457–472.

[34] Stamatakis A. RAxML Version 8: A tool for Phylogenetic analysis and post-analysis of large phylogenies. Bioinformatics. 2014;30: 1312–1313. doi: 10.1093/bioinformatics/btu033 24451623 10.1093/bioinformatics/btu033 PMC3998144

[35] Miller M, Pfeiffer W, Schwartz T. Creating the CIPRES science gateway for inference of large phylogenetic trees. In: Proceedings of the Gateway Computing Environments Workshop (GCE). New Orleans. 2010. pp. 1–8.

[36] Drummond AJ, Suchard MA, Xie D, Rambaut A. Bayesian phylogenetics with BEAUti and the BEAST 1.7. Mol Biol Evol. 2012;29: 1969–1973. doi: 10.1093/molbev/mss075 22367748 10.1093/molbev/mss075 PMC3408070

[37] Drummond AJ, Ho SYW, Phillips MJ, Rambaut A. Relaxed phylogenetics and dating with confidence. PLoS Biol. 2006;4: 699–710. 10.1371/journal.pbio.0040088 PMC1395354 16683862

[38] Weiss FE, Malabarba LR, Malabarba MC. Phylogenetic relationships of *Paleotetra*, a new characiform fish (Ostariophysi) with two new species from the Eocene-Oligocene of south-eastern Brazil. J Syst Palaeontol. 2012;10: 73–86.

[39] Malabarba MC. Phylogeny of fossil Characiformes and paleobiogeography of the Tremembé Formation, São Paulo, Brazil In: Malabarba LR, Reis RE, Vari RP, Lucena ZS, Lucena CS, editors. Phylogeny and Classification of Neotropical Fishes. Rio Grande do Sul: Edipucrs 1998 pp. 69–84.

[40] Bührnheim CM, Carvalho TP, Malabarba LR, Weitzman SW. A new genus and species of characid fish from the Amazon basin—the recognition of a relictual lineage of characid fishes (Ostariophysi: Cheirodontinae: Cheirodontini). Neotrop Ichthyol. 2008;6: 663–678.

[41] Mirande JM, Jerep FC, Vanegas-Ríos JA. Phylogenetic relationships of the enigmatic *Carlastyanax aurocaudatus* (Eigenmann) with remarks on the phylogeny of the Stevardiinae (Teleostei: Characidae). Neotrop Ichthyol. 2013;11: 747–766.

[42] Cohen KM, Finney SC, Gibbard PL, Fan JX. The ICS International Chronostratigraphic Chart. Episodes. 2014;36: 199–204.

[43] Forest F. Calibrating the Tree of Life: fossils, molecules and evolutionary timescales. Ann Bot. 2009;104: 789–794. doi: 10.1093/aob/mcp192 19666901 10.1093/aob/mcp192 PMC2749537

[44] Rambaut A. FigTree, version 1.3.1. 2009. Available from http://tree.bio.ed.ac.uk/software/figtree/.

[45] Pons J, Barraclough TG, Gomez-Zurita J, Cardoso A, Duran DP, Hazell S, et al Sequence-Based Species Delimitation for the DNA Taxonomy of Undescribed Insects. Syst Biol. 2006;55: 595–609. 16967577 10.1080/10635150600852011

[46] Fontaneto D, Herniou E, Boschetti C, Caprioli M, Melone G, Ricci C, et al Independently evolving species in asexual bdelloid rotifers. PLoS Biol. 2007;5: 914–921. 10.1371/journal.pbio.0050087 PMC1828144 17373857

[47] Fujisawa T, Barraclough TG. Delimiting Species Using Single-Locus Data and the Generalized Mixed Yule Coalescent Approach: A Revised Method and Evaluation on Simulated Data Sets. Syst Biol. 2013;62: 707–724. doi: 10.1093/sysbio/syt033 23681854 10.1093/sysbio/syt033 PMC3739884

[48] Costa-Silva GJ, Rodriguez MS, Roxo FF, Foresti F, Oliveira C. Using Different Methods to Access the Difficult Task of Delimiting Species in a Complex Neotropical Hyperdiverse Group. PLoS One. 2015;10: 1–12. 10.1371/journal.pone.0135075 PMC4557985 26332320

[49] R Development Core Team. R: A Language and Environment for Statistical Computing. 2013.

[50] Bandelt HJ, Forster P, Röhl A. Median-joining networks for inferring intraspecific phylogenies. Mol Biol Evol. 1999;16: 37–48. doi: 10.1093/oxfordjournals.molbev.a026036 10331250 10.1093/oxfordjournals.molbev.a026036

[51] Excoffier L, Smouse PE, Quattro JM. Analysis of molecular variance inferred from metric distances among DNA haplotypes: application to human mitochondrial DNA restriction data. Genetics. 1992;131: 479–491. 1644282 10.1093/genetics/131.2.479 PMC1205020

[52] Excoffier L, Lischer HEL. Arlequin suite version 3.5: a new series of programs to perform population genetics analyses under Linux and Windows. Mol Ecol Resour. 2010;10: 564–567. doi: 10.1111/j.1755-0998.2010.02847.x 21565059 10.1111/j.1755-0998.2010.02847.x

[53] Librado P, Rozas J. DnaSP v5: a software for comprehensive analysis of DNA polymorphism data. Bioinformatics. 2009;25: 145–1452. 10.1093/bioinformatics/btp187 19346325

[54] Fu YX. Statistical tests of neutrality of mutations against population growth, hitchhiking and background selection. Genetics. 1997;147: 915–925. 9335623 10.1093/genetics/147.2.915 PMC1208208

[55] Tajima F. Evolutionary relationship of DNA sequences in finite populations. Genetics. 1983;123: 437–460. 10.1093/genetics/105.2.437 PMC1202167 6628982

[56] Ramos-Onsins SE, Rosas J. Statistical Properties of New Neutrality Tests Against Population Growth. Mol Biol Evol. 1992;19: 2092–2100. 10.1093/oxfordjournals.molbev.a004034 12446801

[57] Fink WL, Weitzman SH. The so-called Cheirodontin fishes of Central America with description of two new species (Pisces, Characidae). Smithson Contribu Zool. 1974;172: 1–46.

[58] Menezes NA, Weitzman SH. Two new species of Mimagoniates (Teleostei: Characidae: Glandulocaudinae), their phylogeny and biogeography and a key to the glandulocaudin fishes of Brazil and Paraguay. Proc. Biol. Soc. Wash. 1990;103: 380–426.

[59] Taylor WR, Van Dyke GC. Revised procedures for staining and clearing smallfishes and other vertebrates for bone and cartilage study. Cybium. 1985;9: 107–119.

[60] Weitzman SH, Malabarba LR. Systematics of *Spintherobolus* (Teleostei: Characidae: Cheirodontinae) from Eastern Brazil. Ichthyol. Explor. Freshw. 1999;10: 1–43.

[61] R Development Core Team. Writing R Extensions. Manual included with R version 2.10.0. 2009.

[62] Reid NM, Carstens BC. Phylogenetic estimation error can decrease the accuracy of species delimitation: a Bayesian implementation of the general mixed Yule-coalescent model. BMC Evol Biol. 2012;196: 1–11. 10.1186/1471-2148-12-196 PMC3503838 23031350

[63] Kekkonen M, Hebert PDN. DNA barcode-based delineation of putative species: efficient start for taxonomic workflows. Mol Ecol Resour. 2014;14: 706–15. doi: 10.1111/1755-0998.12233 24479435 10.1111/1755-0998.12233 PMC4264940

[64] Roxo FF, Ochoa LE, Costa-Silva GJ, Oliveira C. Species delimitation in *Neoplecostomus* (Siluriformes: Loricariidae) using morphologic and genetic approaches. DNA Barcodes. 2015;3: 110–117.

[65] Melo BF, Ochoa LE, Vari RP, Oliveira C. Cryptic species in the Neotropical fish genus *Curimatopsis* (Teleostei, Characiformes). Zool. Scr. 2016;45: 650–658.

[66] Razkin O, Gómez-Moliner BJ, Vardinoyannis K, Martínez-Ortí A, Madeira MJ. Species delimitation for cryptic species complexes: case study of *Pyramidula* (Gastropoda, Pulmonata). Zool. Scr. 2017;46: 55–72.

[67] Lohse KD. Can mtDNA Barcodes Be Used to Delimit Species? A Response to Pons et al. (2006). Syst Biol. 2009;48: 439–442. 10.1093/sysbio/syp039 20525596

[68] Talavera G, Dincä V, Vila R. Factors affecting species delimitations with the GMYC model: insights from a butterfly survey. Methods Ecol Evol. 2013;4: 1101–1110.

[69] Henriques JM, Costa-Silva GJ, Ahikaga FY, Hanner R, Foresti F, Oliveira C. Use of DNA barcode in the identification of fish species from Ribeira de Iguape Basin and coastal rivers from São Paulo State (Brazil). DNA Barcodes. 2015;3: 118–128.

[70] de Queiroz K. The General Lineage Concept of Species, Species Criteria, and the Process of Speciation. A Conceptual Unification and Terminological Recommendations In: Howard DJ, de Queiroz K, editors. Endless forms: Species and speciation. New York: Oxford University Press 1998 pp. 57–75.

[71] Sites JW, Marshall JC. Operational criteria for delimiting species. Annu Rev Ecol Evol Syst. 2004;35: 199–227.

[72] Carstens BC, Pelletier RA, Reid NM, Satler JD. How to fail at species delimitation. Mol Ecol. 2013;22: 4369–4383. doi: 10.1111/mec.12413 23855767 10.1111/mec.12413

[73] Malato G, Shervette VR, Navarrete RA, Valdiviezo JR, Nugra FS, Delgado PC, et al Parallel body shape divergence in the Neotropical fish genus *Rhoadsia* (Teleostei: Characidae) along elevational gradients of the western slopes of the Ecuadorian Andes. PLoS One. 2017;12: 1–28. 10.1371/journal.pone.0179432 PMC5489170 28658255

[74] Knowles LL, Carstens BC. Delimiting species without monophyletic gene trees. Syst Biol. 2007;56: 887–895. doi: 10.1080/10635150701701091 18027282 10.1080/10635150701701091

[75] Leliaert F, Verbruggen H, Wysor B, de Clerck O. DNA taxonomy in morphologically plastic taxa: Algorithmic species delimitation in the *Boodlea* complex (Chlorophyta: Cladophorales). Mol Phylogenet Evol. 2009;53: 122–133. doi: 10.1016/j.ympev.2009.06.004 19524052 10.1016/j.ympev.2009.06.004

[76] Barrett CF, Freudenstein JV. An integrative approach to delimiting species in a rare but widespread mycoheterotrophic orchid. Mol Ecol. 2011;20: 2771–2786. doi: 10.1111/j.1365-294X.2011.05124.x 21569137 10.1111/j.1365-294X.2011.05124.x

[77] Avise JC. Phylogeography: the history and formation of species Cambridge: Harvard University Press; 2000.

[78] Bickford D, Lohman DJ, Sodhi NS, Ng PKL, Meier R, Winkler Ket al. Cryptic species as a window on diversity and conservation. Trends in Ecology & Evolution. 2007;22: 148–155. 17129636 10.1016/j.tree.2006.11.004

[79] Seehausen O, Wagner CE. Speciation in freshwater fishes. Annu Rev Ecol Evol Syst. 2014;45: 621–651.

[80] Cooke GM, Chao NL, Beheregaray LB. Divergent natural selection with gene flow along major environmental gradients in Amazonia: insights from genome scans, population genetics and phylogeography of the characin fish *Triportheus albus*. Mol Ecol. 2012;21: 2410–2427. doi: 10.1111/j.1365-294X.2012.05540.x 22512735 10.1111/j.1365-294X.2012.05540.x

[81] Hirschmann A, Malabarba LR, Thomaz AT, Fagundes NJR. Riverine habitat specificity constrains dispersion in a Neotropical fish (Characidae) along Southern Brazilian drainages. Zool. Scr. 2015;44: 374–382.

[82] Cook BD, Adams M, Unmack PJ, Burrows D, Pusey BJ, Perna C, et al Phylogeography of the mouth-brooding freshwater fish *Glossamia aprion* (Apogonidae) in northern and eastern Australia: historical biogeography and allopatric speciation. Biol J Linn Soc Lond. 2017;1–16.

[83] Moore WS. Inferring Phylogenies from mtDNA Variation: Mitochondrial-Gene Trees Versus Nuclear-Gene Trees. Evolution. 1995;49: 718–726. doi: 10.1111/j.1558-5646.1995.tb02308.x 28565131 10.1111/j.1558-5646.1995.tb02308.x

[84] Martins FM, Domingues MV. Filogeografia In: Carvalho CJB, Almeida EAB, editors. Biogeografia da América do Sul: padrões e processos. São Paulo: Roca 2011 pp. 137–150.

[85] Avise JC, Arnold J, Ball RM, Bermingham E, Lamb T, Neigel JE, et al Intraspecific phylogeography: the mitochondrial DNA bridge between population genetics and systematics. Annu Rev Ecol Evol Syst. 1987;18: 489–522.

[86] Grant WS, Bowen BW. Shallow population histories in deep evolutionary lineages of marine fishes: insights from sardines and anchovies and lessons for conservation. J Heredity.1998;89: 415–426.

[87] Spellman GM, Klicka J. Testing hypotheses of Pleistocene population history using coalescent simulations: phylogeography of the pygmy nuthatch (*Sitta pygmaea*). Proc R Soc Lond B Biol Sci. 2006;73: 3057–3063. 10.1098/rspb.2006.3682 PMC1679902 17015345

[88] Myers GS. Fresh-water fishes and West Indian zoogeography. Annu Rep Smithson Inst. 1938;3465: 339–364.

[89] Vari RP. The Curimatidae, a Lowland Neotropical fish family (Pisces: Characiformes): distribution, endemism, and phylogenetic biogeography In: Vanzolini PE, Heyer WR, editors. Proceedings of a workshop on Neotropical distribution patterns. Rio de Janeiro: Academia Brasileira de Ciências 1998 pp. 343–377.

[90] Albert JS, Petry P, Reis RE. Major biogeographic and phylogenetic patterns In: Albert JS, Reis RE, editors. Historical Biogeography of Neotropical Freshwater Fishes. Berkley: University of California Press 2011 pp. 21–57

[91] Thomaz AT, Malabarba LR, Bonatto SL, Knowles LL. Testing the effect of palaeodrainages versus habitat stability on genetic divergence in riverine systems: study of a Neotropical fish of the Brazilian coastal Atlantic Forest. J Biogeogr. 2015;1–13.

[92] Thomaz AT, Malabarba LR, Knowles LL. Genomic signatures of paleodrainages in a freshwater fish along the southeastern coast of Brazil: genetic structure reflects past riverine properties. Heredity. 2017; 1–8. 10.1038/hdy.2017.46 PMC5597787 28767104

[93] Albert JS, Carvalho TP. Neogene Assembly of Modern Faunas In: Albert JS, Reis RE, editors. Historical Biogeography of Neotropical Freshwater Fishes. Berkley: University of California Press 2011 pp. 119–136.

[94] Small RJ. The Study of Landforms: a Textbook of Geomorphology. Cambridge: Cambridge University Press; 1977.

[95] Bishop P. Drainage rearrangement by river capture, beheading and diversion. Prog Phys Geogr. 1995;19: 449–47.

[96] Dagosta FCP, de Pinna M. Biogeography of Amazonian fishes: deconstructing river basins as biogeographic units. Neotrop Ichthyol. 2017;15: 1–24. doi: 10.1590/1982-0224-20170034

[97] Roxo FF, Zawadzki CH, Alexandrou MA, Costa-Silva GJ, Chiachio MC, Foresti F, et al Evolutionary and biogeographic history of the subfamily Neoplecostominae (Siluriformes: Loricariidae). Ecol Evol. 2012;2: 2438–2449. doi: 10.1002/ece3.368 23145330 10.1002/ece3.368 PMC3492771

[98] Ab’Saber AN. O problema das conexões antigas e da separação da drenagem do Paraíba e Tietê. Boletim Paulista de Geografia. 1957;26: 38–49.

[99] Saadi A. A geomorfologia da Serra do Espinhaço em Minas Gerais e de suas margens. Geonomos. 1998;3: 41–63.

[100] Costa WJEM. The neotropical annual fish genus *Cynolebias* (Cyprinodontiformes: Rivulidae): phylogenetic relationships, taxonomic revision and biogeography. Ichthyol. Explor. Freshw. 2001;12: 333–383.

[101] Oliveira D. A captura do Alto Rio Guaratuba: uma proposta metodológica para o estudo da evolução do relevo na Serra do Mar, Boracéia-SP. Ph.D. Thesis, Universidade de São Paulo. 2003. Available from: http://www.teses.usp.br/teses/disponiveis/8/8135/tde-05052004-134328/pt-br.php

[102] Oliveira D. Capturas fluviais como evidências da evolução do relevo: uma revisão bibliográfica. Revista do Departamento de Geografia 2010;20: 37–50.

[103] Riccomini C, Sant’Anna LG, Ferrari AL. Evolução geológica do Rift Continental do Sudeste do Brasil In: Mantesso-Neto V, Bartorelli A, Carneiro CDR, Brito-Neves BB, editors. Geologia do continente Sul-Americano: evolução da obra de Fernando Flávio Marques de Almeida. São Paulo: Editora Beca 2004 pp. 383–405.

[104] Oliveira D, Neto JPQ. Estudo da evolução do relevo na Serra do Mar no Estado de São Paulo a partir de um caso de captura fluvial. Geousp. 2007;22: 73–88.

[105] Souza CRG. The Bertioga Coastal Plain: An Example of Morphometric Evolution In: Vieira BC, Salgado AAR, Santos LJC, editors *Landscapes and Landforms of Brazil*. New York: Springer Verlag 2015 pp. 115–134.

[106] Jacintho LRC. Geoprocessamento e sensoriamento remoto como ferramentas na gestão ambiental de Unidades de Conservação: o caso da Área de Proteção Ambiental (APA) do Capivari-Monos, São Paulo—SP. Ph.D. Thesis, Universidade de São Paulo. 2003. Available from: http://www.teses.usp.br/teses/disponiveis/44/44133/tde-14082003-230137/pt-br.php

[107] Ab'saber AN. Geomorfologia do sítio urbano de São Paulo. São Paulo: Ateliê Editorial; 2007.

[108] Marinho MMF, Dagosta FCP, Birindelli JLO. *Hemigrammus ataktos*: a new species from the rio Tocantins basin, central Brazil (Characiformes: Characidae). Neotrop Ichthyol. 2014;12: 257–264.

[109] Menezes NA. Família Characidae: Glandulocaudinae In: Buckup PA, Menezes NA, Ghazzi MS, editors. Catálogo das espécies de peixes de água doce do Brasil. Rio De Janeiro: Museu Nacional 2007 pp. 38–39.

[110] Aguirre WE, Navarrete R, Malato G, Calle P, Loh MK, Vital WF, et al Body Shape Variation and Population Genetic Structure of *Rhoadsia altipinna* (Characidae: Rhoadsiinae) in Southwestern Ecuador. Copeia. 2016;104: 554–569.

[111] Zamudio KL, Robertson JM, Chan LM, Sazima I. Population structure in the catfish *Trichogenes longipinnis*: drift offset by asymmetrical migration in a tiny geographic range. Biol J Linn Soc Lond. 2009;97: 259–274.

[112] IUCN. IUCN Red List Categories and Criteria: Version 3.1. IUCN Species Survival Commission. Switzerland and Cambridge: Gland 2001.

[113] Menezes NA, Lima FCT.*landulocauda melanogenys* Eigenmann, 1911 In: Machado ABM, Drumond GM, Paglia AP, editors. *Livro vermelho da fauna brasileira ameaçada de extinção*. Minas Gerais: Fundação Biodiversitas 2008 pp. 62–63

[114] Peña J, Barnos S, Bobo-Pinilla J, Lorite J, Martínez-Ortega MM. Designing conservation strategies to preserve the genetic diversity of *Astragalus edulis* Bunge, an endangered species from western Mediterranean region. PeerJ. 2016;1–20. doi: 10.7717/peerj.1474 26844014 10.7717/peerj.1474 PMC4736990

[115] Menezes NA, Weitzman SH, Oyakawa OT, Lima FCT, Castro RMC, Weitzman MJ. Peixes de água doce da Mata Atlântica: lista preliminar de espécies e comentários sobre conservação de peixes de água doce neotropicais São Paulo: Museu de Zoologia da Universidade de São Paulo; 2007.

