# Supplementary material for: Molecular and morphological data of the freshwater fish Glandulocauda melanopleura (Characiformes: Characidae) provide evidences of river captures and local differentiation in the Brazilian Atlantic Forest
Source: PLoS One. 2018 Mar 26;13(3):e0194247. doi: 10.1371/journal.pone.0194247 (PMC5868800; doi:10.1371/journal.pone.0194247)
Supplement: S1 Appendix — (DOCX) [file pone.0194247.s001.docx]

**S1 Appendix. Material examined of *Glandulocauda melanopleura* and *G. caerulea* in the morphological analyses.** Collections abbreviations follow Fricke & Eschmeyer (2017). Catalog numbers are followed by the total number of specimens in alcohol, standard length (SL) range of all specimens of the lot, and, if any, the number of cleared and stained specimens (c&s) and/or the presence of samples of tissue (mol).

***Glandulocauda melanopleura.*** All from Brazil, São Paulo state. Rio Paraná basin/Upper Rio Tietê basin: LBP 4507, 10, 40.5-45.0 mm SL, 7 mol, Santo André, Rio Paranapiacaba, near to village of Paranapiacaba; MZUSP 26891, 3, 43.4-52.3 mm SL, Santo André, Reserva Biológica de Paranapiacaba; MZUSP 28849, 10, 26.9-32.7 mm SL, Santo André, stream tributary of Rio Grande, Campo Grande; MZUSP 35242, 8, 1 c&s, 33.8-39.1 mm SL, Santo André, stream near to Paranapiacaba; MZUSP 74333, 10, 1 c&s, 25.2-30.2 mm SL, Paranapiacaba, dirt road between Campo Grande-Paranapiacaba; MZUSP 86967, 3, 43.6-57.5 mm SL, Santo André, stream at Reserva Biológica do Alto da Serra de Paranapiacaba; MZUSP 86984, 2, 24.9-44.0 mm SL, Santo André, Rio Pardo, tributary of Rio Grande, at dirt road between Campo Grande-Paranapiacaba. Rio Itanhaém basin/Rio Branco sub-basin: MZUSP 108577, 2, 29.7-36.2 mm SL, São Paulo, Rio Capivari, at Parque Estadual da Serra do Mar (Núcleo Curucutu); MZUSP 108621, 8, 24.3-31.9 mm SL, São Paulo, Rio Capivari, at APA Capivari-Monos; MZUSP 108724, 1, 54.0 mm SL, São Paulo, Rio Capivari, near the entrance to the Parque Estadual da Serra do Mar (Núcleo Curucutu), at APA Capivari-Monos; MZUSP 111017, 22, 2 c&s, 10 mol, 14.5-57.4 mm SL, Itanhaém, stream tributary of Rio Capivari, at APA Capivari-Monos. Rio Guaratuba basin: MZUSP 84412, 10, 19.2-37.2 mm SL, Bertioga, stream tributary of rio Guaratuba, Trilha do Mirante, Estação Biológica de Boracéia; MZUSP 87567, 23, 18.2-36.1 mm SL, same locality as MZUSP 84412; MZUSP 87571, 43, 1 d&c, 29.5-44.0 mm SL, Bertioga, stream tributary of Rio Guaratuba, at road of Estação Elevatória, Estação Biológica de Boracéia; MZUSP 115244, 20, 12 mol, 33.0-39.4 mm SL, stream tributary of Rio Coruja, at road of Estação Elevatória, Estação Biológica de Boracéia**.** Rio Ribeira de Iguape basin: MZUSP 79429, 3, 37.5-48.9 mm SL, Juquitiba, stream tributary of rio Juquiá flowing into rio Ribeira drainage, fazenda Santa Rita. Rio Itatinga basin**:** DZSJRP 6613, 2, 26.2-26.6 mm SL, Bertioga, stream tributary of right bank of the Rio Itatinga. ***Glandulocauda caerulea*.** All from Brazil, Paraná state, Rio Paraná basin/Upper Rio Iguaçu basin. MRRJ 19537, 5, 34.4-40.8 mm SL, Quatro Barras, stream tributrary of rio Iraí, within the property of Mr. Renato Venske; MZUSP 97663, 5, 21.9-40.8 mm SL, Quatro Barras, stream tributary of Rio Iraí; MZUSP 97664, 5, 26.6-41.8 mm SL, same locality as MZUSP 97663; MZUSP 97665, 2, 30.1-46.5, same locality as MZUSP 97663; MZUSP 97666, 3, 1 c&s, 34.3-38.7 mm SL, Pinhais, stream tributary of Rio Palmital; MZUSP 117479, 4, 4 mol, 28.9-34.1 mm SL, Balsa Nova, stream tributary of Ribeirão Amola Faca, near Fazenda Lara Maria.
