# Supplementary figures and images for: Molecular and morphological data of the freshwater fish Glandulocauda melanopleura (Characiformes: Characidae) provide evidences of river captures and local differentiation in the Brazilian Atlantic Forest
Source: PLoS One. 2018 Mar 26;13(3):e0194247. doi: 10.1371/journal.pone.0194247 (PMC5868800; doi:10.1371/journal.pone.0194247)

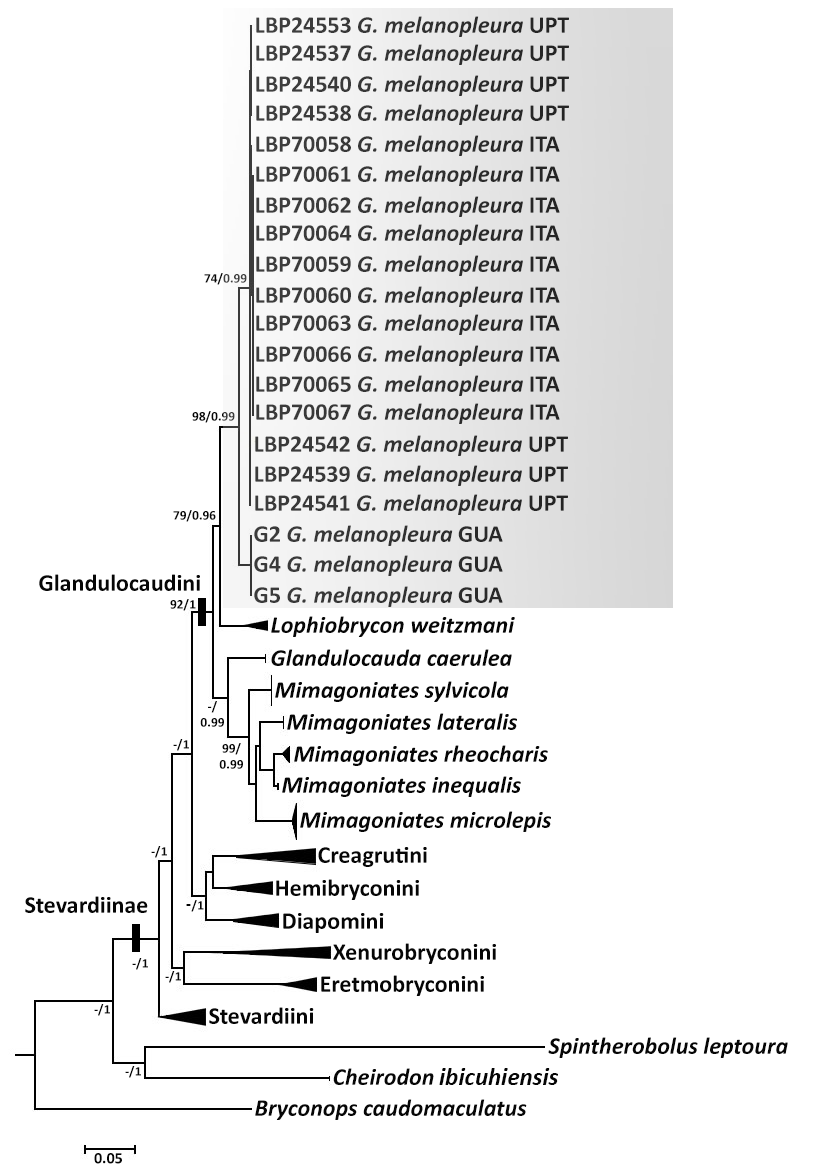

Supplement: S1 Fig — In highlight, the placement of the species within Glandulocaudini and relationships among three populations of this species: upper Rio Tietê (UPT), Rio Itanhaém (ITA), and Rio Guaratuba (GUA). Numbers at branches are bootstrap values from 1,000 bootstrap pseudoreplicates obtained from ML analysis and posterior probabilities obtained in the Bayesian Inference analysis. Values below 70% and 0.90 (–) are not shown. (TIF) [file pone.0194247.s002.tif]

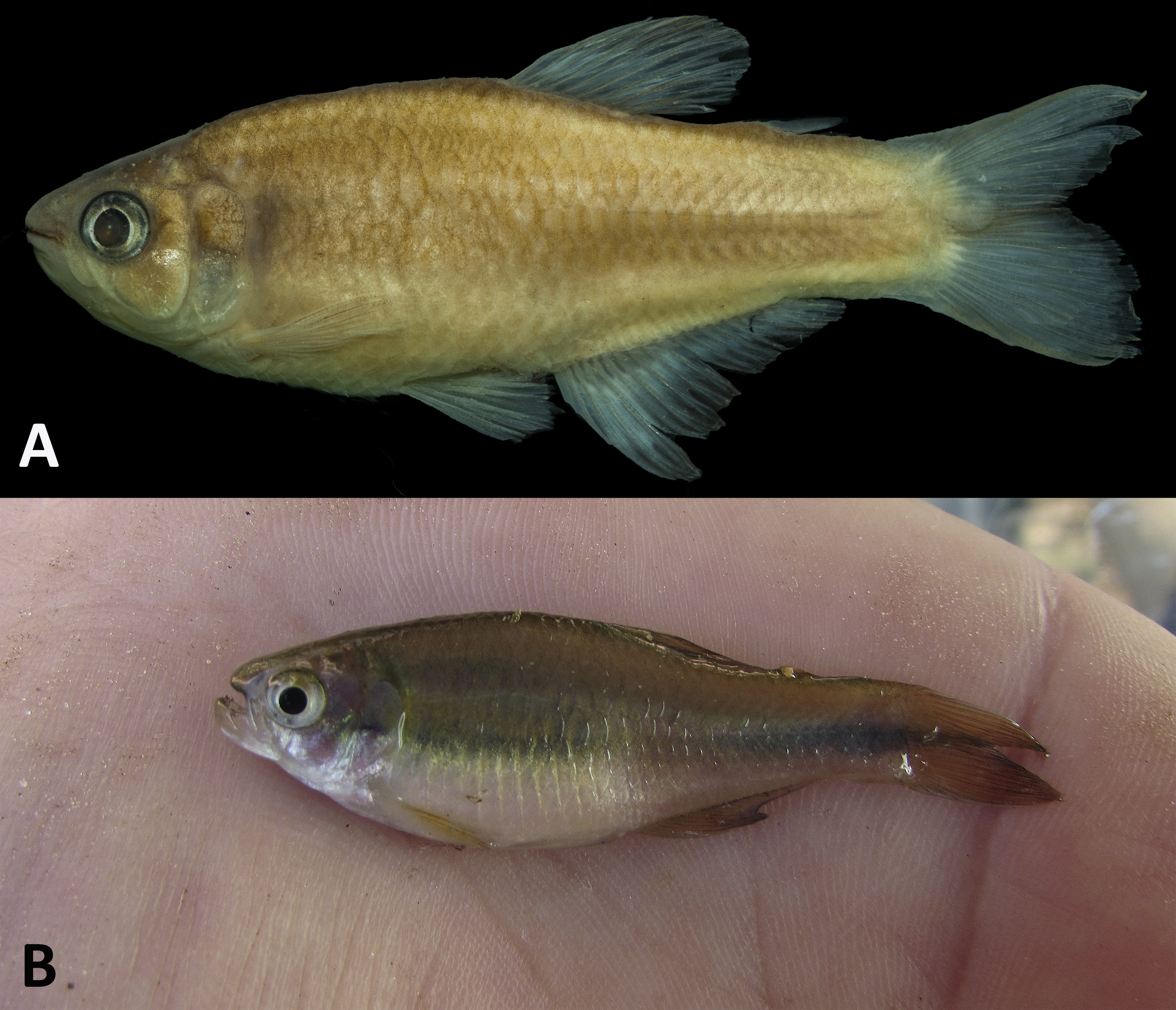

Supplement: S2 Fig — All from upper Rio Iguaçu basin, Paraná state, Brazil. (A) fixed specimen, MZUSP 97663, male, 40.8 mm SL and (B) alive specimen, MZUSP 117479, male, 34.1 mm SL. (TIF) [file pone.0194247.s003.tif]
