# Supplementary material for: Molecular and morphological data of the freshwater fish Glandulocauda melanopleura (Characiformes: Characidae) provide evidences of river captures and local differentiation in the Brazilian Atlantic Forest
Source: PLoS One. 2018 Mar 26;13(3):e0194247. doi: 10.1371/journal.pone.0194247 (PMC5868800; doi:10.1371/journal.pone.0194247)
Supplement: S1 Table — (DOCX) [file pone.0194247.s006.docx]

**S1 Table. List of lot number, voucher, and GenBank accession number for each taxon used in this study (with exception of *Glandulocauda melanopleura*).** Collections abbreviations follow Fricke & Eschmeyer (2017), except for GO (= Guillermo Ortí laboratory collection) and MZict (= Tissue collection of Museu de Zoologia da Universidade de São Paulo).

| **Taxon** | **Lot number** | | | **Voucher** | **GenBank accession number (16S/COI)** | |
| --- | --- | --- | --- | --- | --- | --- |
| **Stevardiin species** |  | | |  |  |  |
| **Glandulocaudini** |  | | |  |  |  |
| *Lophiobrycon weitzmani* | LBP 8166 | | | LBP 38090 | HQ171411/ GU701436 |  |
| *Lophiobrycon weitzmani* | MNRJ 31626 | | | MNRJ 31626 | KF209982/ KF210232 |  |
| *Lophiobrycon weitzmani* | MNRJ 31664 | | | MNRJ 31664 | KF209983/ KF210233 |  |
| *Glandulocauda caerulea* | MZUSP 117479 | | | MZict 2612 | MG958090/ MG967567 |  |
| *Glandulocauda caerulea* | MZUSP 117479 | | | Mzict 2613 | MH036165/ MH036055 |  |
| *Glandulocauda caerulea* | MZUSP 117479 | | | MZict 2614 | MH036164/ MH036054 |  |
| *Glandulocauda caerulea* | MZUSP 117479 | | | MZict 2615 | MH036166/ MH036056 |  |
| *Mimagoniates inequalis* | LBP 3383 | | | LBP 21275 | MG953575/ MG967570 |  |
| *Mimagoniates inequalis* | LBP 3383 | | | LBP 21277 | MG953576/ MG967571 |  |
| *Mimagoniates inequalis* | LBP 3383 | | | LBP 21278 | MH036129/ MH036057 |  |
| *Mimagoniates lateralis* | LBP 8212 | | | LBP 38201 | MH036118/ MH036059 |  |
| *Mimagoniates lateralis* | LBP 8212 | | | LBP 38431 | MH036119/ MH036060 |  |
| *Mimagoniates lateralis* | LBP 70078 | | | | MH036120/ MH036061 |  |
| *Mimagoniates lateralis* | LBP 70079 | | | | MH036121/ MH036062 |  |
| *Mimagoniates lateralis* | LBP 70080 | | | | MH036117/ MH036058 |  |
| *Mimagoniates lateralis* | LBP 70082 | | | | MG953578/ MG967574 |  |
| *Mimagoniates microlepis* | LBP 10756 | | | LBP 49795 | MG953584/ MG967609 |  |
| *Mimagoniates microlepis* | LBP 10756 | | | LBP 49796 | MH036132/ MH036066 |  |
| *Mimagoniates microlepis* | LBP 10756 | | | LBP 49797 | MH036133/ MH036067 |  |
| *Mimagoniates microlepis* | LBP 10756 | | | LBP 49798 | MH036134/ MH036068 |  |
| *Mimagoniates microlepis* | LBP 10756 | | | LBP 49799 | MH036135/ MH036069 |  |
| *Mimagoniates microlepis* | LBP 14362 | | | LBP 54765 | MH036136/ MH036070 |  |
| *Mimagoniates microlepis* | MZUSP 115095 | | | LBP 70084 | MH036139/ MH036073 |  |
| *Mimagoniates microlepis* | MZUSP 115095 | | | LBP 70085 | MH036140/ MH036074 |  |
| *Mimagoniates microlepis* | MZUSP 115095 | | | LBP 70086 | MH036141/ MH036075 |  |
| *Mimagoniates microlepis* | LBP 70088 | | | | MH036142/ MH036076 |  |
| *Mimagoniates microlepis* | LBP 70089 | | | | MH036143/ MH036077 |  |
| *Mimagoniates microlepis* | LBP 70090 | | | | MH036144/ MH036078 |  |
| *Mimagoniates microlepis* | LBP 70091 | | | | MH036145/ |  |
| *Mimagoniates microlepis* | LBP 70092 | | | | MH036138/ MH036072 |  |
| *Mimagoniates microlepis* | MZUSP 118711 | | | MZict 2616 | MG953589/ MG967603 |  |
| *Mimagoniates microlepis* | MZUSP 118711 | | | MZict 2617 | MH036137/ MH036071 |  |
| *Mimagoniates microlepis* | MZUSP 118711 | | | MZict 2618 | MH036130/ MH036063 |  |
| *Mimagoniates microlepis* | MZUSP 118711 | | | MZict 2619 | MH036131/ MH036064 |  |
| *Mimagoniates sylvicola* | LBP 70003 | | | | MH036102/ MH036079 |  |
| *Mimagoniates sylvicola* | LBP 70004 | | | | MH036103/ MH036080 |  |
| *Mimagoniates sylvicola* | LBP 70005 | | | | MH036104/ MH036081 |  |
| *Mimagoniates sylvicola* | LBP 70006 | | | | MH036105/ MH036082 |  |
| *Mimagoniates sylvicola* | LBP 70007 | | | | MH036106/ MH036083 |  |
| *Mimagoniates sylvicola* | LBP 70008 | | | | MH036107/ MH036084 |  |
| *Mimagoniates sylvicola* | LBP 70009 | | | | MH036108/ MH036085 |  |
| *Mimagoniates sylvicola* | LBP 70010 | | | | MH036109/ MH036086 |  |
| *Mimagoniates sylvicola* | LBP 70011 | | | | MH036110/ MH036087 |  |
| *Mimagoniates sylvicola* | LBP 70012 | | | | MH036111/ MH036088 |  |
| *Mimagoniates sylvicola* | LBP 70013 | | | | MH036112/ MH036089 |  |
| *Mimagoniates sylvicola* | LBP 70014 | | | | MH036113/ MH036090 |  |
| *Mimagoniates sylvicola* | LBP 70015 | | | | MH036114/ MH036091 |  |
| *Mimagoniates sylvicola* | LBP 70016 | | | | MH036115/ MH036092 |  |
| *Mimagoniates sylvicola* | LBP 70017 | | | | MH036116/ MH036093 |  |
| *Mimagoniates rheocharis* | MCP 28770 | | | | MH036123/ MH036095 |  |
| *Mimagoniates rheocharis* | UFRGS 12896 | TEC 911A | | | MH036122/ MH036094 |  |
| *Mimagoniates rheocharis* | UFRGS 12896 | TEC 911C | | | MG953590/ MG967572 |  |
| *Mimagoniates rheocharis* | UFRGS 16561 | TEC 2893A | | | MH036124/ MH036096 |  |
| *Mimagoniates rheocharis* | UFRGS 16561 | TEC 2893B | | | MH036128/ MH036100 |  |
| *Mimagoniates rheocharis* | UFRGS 12588 | TEC 1264A | | | MH036125/ MH036097 |  |
| *Mimagoniates rheocharis* | UFRGS 12588 | TEC 1264B | | | MH036126/ MH036098 |  |
| *Mimagoniates rheocharis* | UFRGS 12588 | TEC 1264C | | | MH036127/ MH036099 |  |
| **Creagrutini** |  | | | |  |  |
| *Carlastyanax aurocaudatus* | GO 001 | | | | KF209782/ KF210087 |  |
| *Carlastyanax aurocaudatus* | GO 002 | | | | KF209783/ KF210088 |  |
| *Carlastyanax aurocaudatus* | GO 004 | | | | KF209784/ KF210089 |  |
| *Creagrutus barrigai* | MUSM 33815 | | | | KF209800/ KF210103 |  |
| *Creagrutus barrigai* | MUSM 39470 | AP 87 | | | KF209801/ KF210104 |  |
| **Diapomini** |  |  | | |  |  |
| *Diapoma terofali* | UFRGS 12891 | TEC 339A | | | KF209854/ KF210143 |  |
| *Diapoma terofali* | UFRGS 12892 | TEC 392 | | | KF209855/ KF210144 |  |
| *Piabina argentea* | MCP 42401 | | | | KF210001/ KF210250 |  |
| *Piabina argentea* | MNRJ 31758 | 987 | | | KF210002/ KF210251 |  |
| *Piabina argentea* | UFRGS 11373 | TEC 1211A | | | KF210003/ KF210252 |  |
| *Piabina argentea* | UFRGS 12887 | TEC 1032 | | | KF210004/ KF210253 |  |
| **Eretmobryconini** |  | | | |  |  |
| *Bryconamericus dahli* | STRI 9302 | | | | KF209727/ KF210052 |  |
| *Bryconamericus dahli* | STRI 9308 | | | | KF209728/ KF210053 |  |
| *Bryconamericus dahli* | STRI 9567 | | | | KF209729/ KF210054 |  |
| *Bryconamericus emperador* | STRI 3936 | | | | KF209733/ KF210058 |  |
| *Bryconamericus emperador* | STRI 861 | | | | KF209735/ KF210059 |  |
| **Hemibryconini** |  | | | |  |  |
| *Acrobrycon ipanquianus* | ANSP 180771 | | | | KF209698/ KF210030 |  |
| *Acrobrycon ipanquianus* | ANSP 180776 | | | | KF209699/ KF210031 |  |
| *Hemibrycon huambonicus* | MHNG 2731.041 | | | 152.31 | KF209913/ KF210174 | |
| *Hemibrycon huambonicus* | MHNG 2731.041 | | | 152.32 | KF209914/ KF210175 | |
| *Hemibrycon huambonicus* | MHNG 2731.041 | | | 152.33 | KF209915/ KF210176 | |
| **Stevardiini** |  | | | |  |  |
| *Chrysobrycon myersi* | MHNG 2731.033 | | 152.1 | | KF209791/ KF210095 |  |
| *Chrysobrycon myersi* | MHNG 2731.033 | | 152.9 | | KF209790/ KF210096 |  |
| *Pseudocorynopoma doriae* | MCP 21286 | | | | KF210011/ KF210260 |  |
| *Pseudocorynopoma doriae* | UFRGS 12361 | | TEC 621 | | KF210012/ KF210261 |  |
| *Pseudocorynopoma doriae* | UFRGS 12389 | | TEC 693A | | KF210013/ KF210262 |  |
| **Xenurobryconini** |  | | | |  |  |
| *Tyttocharax tambopatensis* | UFRGS 12884 | | TEC 1594 | | KF210023/ KF210270 |  |
| *Xenurobrycon polyancistrus* | UFRGS 12845 | | TEC 155A | | KF210028/ KF210275 |  |
| *Xenurobrycon polyancistrus* | UFRGS 12845 | | TEC 155B | | KF210029/ KF210276 |  |
| **Non-stevardiin species** |  | | | |  |  |
| *Bryconops caudomaculatus* | UFRGS 12678 | | TEC 1415 | | KF209861/ KF210145 |  |
| *Cheirodon ibicuhiensis* | UFRGS 12508 | | TEC 1326A | | KF209865/ KF210149 |  |
| *Cheirodon ibicuhiensis* | UFRGS 12508 | | TEC 1326B | | KF209866/ KF210150 |  |
| *Spintherobolus leptoura* | LBP 7544 | | LBP 36098 | | HQ171393/ MH036101 |  |
